# 3D tissue model to patient atlas: dynamic network topology reveals PDAC chemoresistance bottlenecks

**DOI:** 10.1038/s41540-026-00830-z

**Published:** 2026-10-23

**Authors:** Maryam Almasi, Jesús Guillermo Nieves Pereira, Thomas Dandekar, Gudrun Dandekar, Johannes Balkenhol

**Affiliations:** 1 Department of Bioinformatics, Biocenter, University of Würzburg, Am Hubland, Würzburg, Germany; 2 Department for Functional Materials in Medicine and Dentistry, University Hospital Würzburg, Würzburg, Germany; 3 Section for Molecular Medicine of Signal Transduction, Institute of Molecular Medicine, Faculty of Medicine, Martin Luther University Halle-Wittenberg, 06120 Halle (Saale), Germany

**Keywords:** Cancer, Computational biology and bioinformatics, Oncology

## Abstract

Pancreatic ductal adenocarcinoma (PDAC) develops chemoresistance through dynamic cell-state transitions, yet the regulatory network architecture governing drug-tolerant states remains poorly characterised, because static expression snapshots cannot capture how interaction topology evolves along treatment-induced trajectories. We established a 3D PANC-1 tissue model on decellularised porcine intestinal matrix, which reaches patient-like proliferation indices and tissue-niche-specific drug responses, and performed single-cell RNA sequencing across four conditions: control, gemcitabine (GEM), TGF-β1, and their combination. Coupling pseudotime trajectory inference to dynamic protein–protein interaction network analysis, we tracked betweenness centrality continuously across cell-state transitions rather than comparing discrete states. GEM survivors engaged a proliferative programme (E2F1, CDK1, AURKA, TPX2, TOP2A, BIRC5), whereas TGF-β1 induced an independent EMT attractor (SPOCK1, MBOAT2, COL5A1, THBS1, FN1). Where the two converged, an emergent bottleneck appeared: CDK1 betweenness centrality spiked selectively, restrained by CDKN1A (p21) and WEE1, defining an S-phase-persistence state enriched for GEM survivors. Cross-examination in a PDAC atlas of 726,107 cells from 231 patients showed 8.7-fold metastatic enrichment of triple-positive cells. This framework, linking controlled 3D tissue experimentation, single-cell systems analysis and clinical-scale patient data, is applicable to drug resistance across cancer types.

**Figure:**
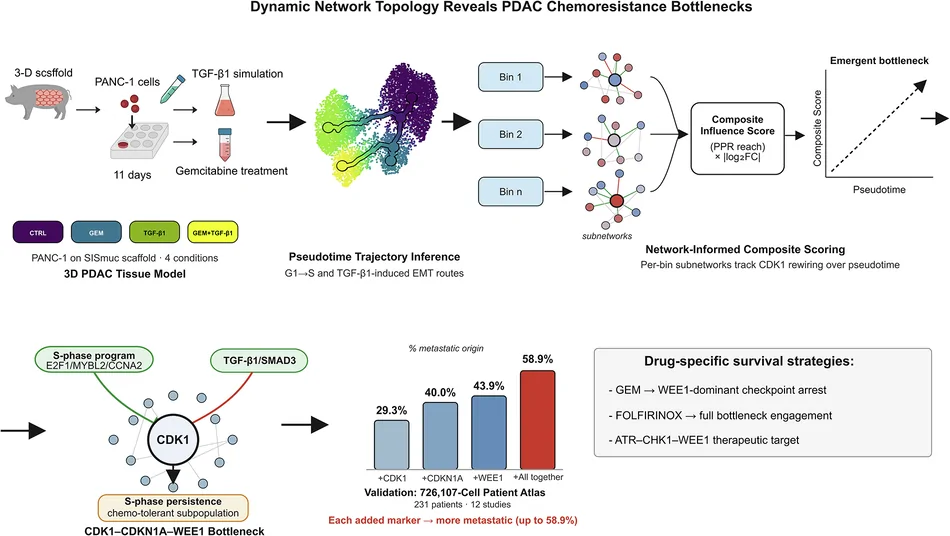

## Introduction

Pancreatic ductal adenocarcinoma (PDAC) is characterised by exceptionally high chemoresistance and mortality^1^. Late diagnosis limits the success of surgical treatment, and standard chemotherapy, gemcitabine (GEM) alone or combined with fluoropyrimidine-based regimens, yields a median overall survival of approximately one year^2–4^. Adjuvant FOLFIRINOX shows improved efficacy over GEM monotherapy but carries a less favourable safety profile^5,6^.

GEM is a nucleoside analogue in clinical use for over 25 years. Resistance arises through multiple mechanisms including poor tumour penetration due to dense stromal content, impaired cellular uptake via human nucleoside transporters, and incomplete cell-cycle arrest—checkpoints remain delayed even after drug removal^7–9^. This suggests that dynamic cell-state transitions under drug pressure, rather than static expression differences alone, govern which cells survive treatment. Resolving these transitions requires both a physiologically relevant experimental platform and an analytical framework capable of tracking regulatory architecture continuously across states, neither of which is achievable in conventional 2D culture or with standard differential expression approaches.

We address the experimental requirement with a 3D intestinal matrix-based tumour model (SISmuc: Small Intestinal Submucosa with preserved mucosa) harbouring PANC-1 cells carrying common PDAC driver mutations KRAS, TP53, and CDKN2A^10^. Unlike 2D cultures where artificially high proliferation and absent tissue architecture preclude physiological drug responses, our 3D model reaches a homeostatic state after ~11 days with proliferation indices (~35% Ki-67-positive) closely matching the clinical mean in PDAC patients^11–13^, enabling single-cell dissection of chemoresistance under near-physiological conditions. Critically, the 3D tissue model and single-cell RNA sequencing are complementary rather than alternative approaches: the model provides the physiological tissue context and ECM architecture absent in 2D cultures, while scRNA-seq provides the single-cell resolution needed to resolve individual cell states and their transitions within that context. The preserved ECM architecture, including distinct crypt and villus niches, enables tissue-niche-specific GEM responses. To recapitulate the desmoplastic reaction characteristic of PDAC^9^, PANC-1 models were additionally stimulated with TGF-β1, generating four experimental conditions: control, GEM, TGF-β1, and GEM + TGF-β1.

Building on companion pseudo-bulk analysis of this dataset that identified differently expressed genes in GEM treated and untreated cells^14^, we complement that work at single-cell resolution by integrating pseudotime trajectory inference with dynamic protein–protein interaction (PPI) network analysis. Rather than comparing discrete treated-versus-untreated snapshots, this framework continuously tracks how network topology, specifically which gene on protein-level act as communication bottlenecks shifts as cells traverse treatment-induced state transitions. This resolves the regulatory decision points that drive GEM resistance rather than merely marking its transcriptional correlates.

To determine whether the regulatory architecture identified in our controlled 3D system reflects human tumour biology and thereby validate both the experimental model and the analytical framework simultaneously, we cross-examined key signatures in a comprehensive PDAC single-cell atlas comprising 726,107 cells from 231 patients across 12 independent studies^15^. Concordance between 3D tissue model findings and patient-scale single-cell data would confirm that the model captures clinically relevant biology and that the network topology approach extracts generalisable mechanistic insight. The resulting framework identifies the CDK1–CDKN1A–WEE1 axis as a chemoresistance bottleneck and highlights intervention points relevant to ATR–CHK1–WEE1 and TGF-β1/SMAD3 signalling axes currently under investigation in PDAC clinical trials.

## Results

### 3D PANC-1 tissue model reveals spatial and transcriptional heterogeneity underlying gemcitabine resistance

**Fig. 1:**
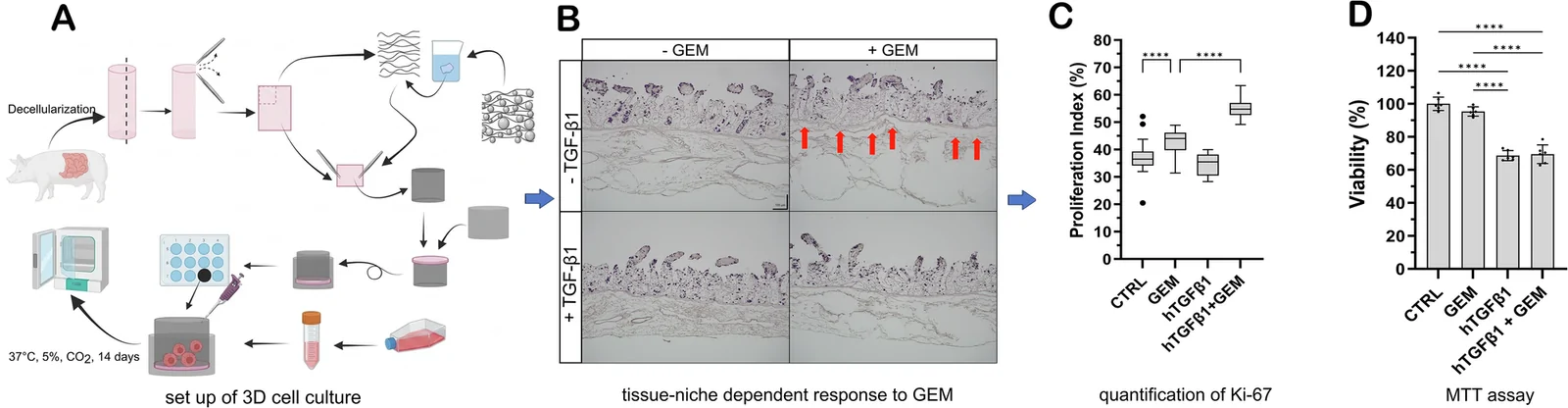
Integrated workflow for dissecting gemcitabine resistance in 3D PDAC cultures. **A** Schematic overview of the experimental workflow. 3D PANC-1 cultures were established on decellularized porcine intestinal matrix (SISmuc: Small Intestinal Submucosa with preserved mucosa), which provides a physiological ECM scaffold with distinct crypt and villus niches. After 11 days, cultures reach a homeostatic proliferation index (~35% Ki-67 positive) closely matching the clinical mean in PDAC patients. Four experimental conditions were generated (control, GEM 10 µM 24 h, TGF-β1 10 ng/mL days 3–14, combined GEM + TGF-β1), followed by single-cell dissociation, scRNA-seq library preparation (10x Genomics 3′ v3), and the integrated computational analysis pipeline shown. **B** H&E staining reveals spatial heterogeneity in GEM sensitivity (10 µM, 24 h): tumor cells in crypt niches are preferentially eradicated (red arrows), while TGF-β1-stimulated cells (11 days) invade across the preserved basement membrane. Scale bar: 100 µm. **C** Ki-67 quantification (5 images per condition) shows increased proliferation index after GEM treatment, further enhanced by TGF-β1 co-stimulation (*n* = 3; ****p < 0.0001). Error bars: SD. **D** MTT viability assay demonstrates high overall chemoresistance in the 3D model; TGF-β1 reduces metabolic activity but GEM produces no additional change at the population level (*n* = 3; ****p < 0.0001, *p < 0.05). Error bars: SD.

To investigate GEM resistance in a physiologically relevant context, we established 3D PANC-1 tissue models on decellularized porcine intestinal matrix (SISmuc), which recapitulate native tissue architecture and patient-like proliferation indices (Fig. 1A). H&E imaging revealed marked spatial heterogeneity: GEM-sensitive cells were preferentially eradicated in crypt niches, while Ki-67 quantification showed that proliferative survivors were further enriched by TGF-β1 co-stimulation (Fig. 1B–C). MTT viability confirmed high overall chemoresistance of the 3D population (Fig. 1D).

**Fig. 2:**
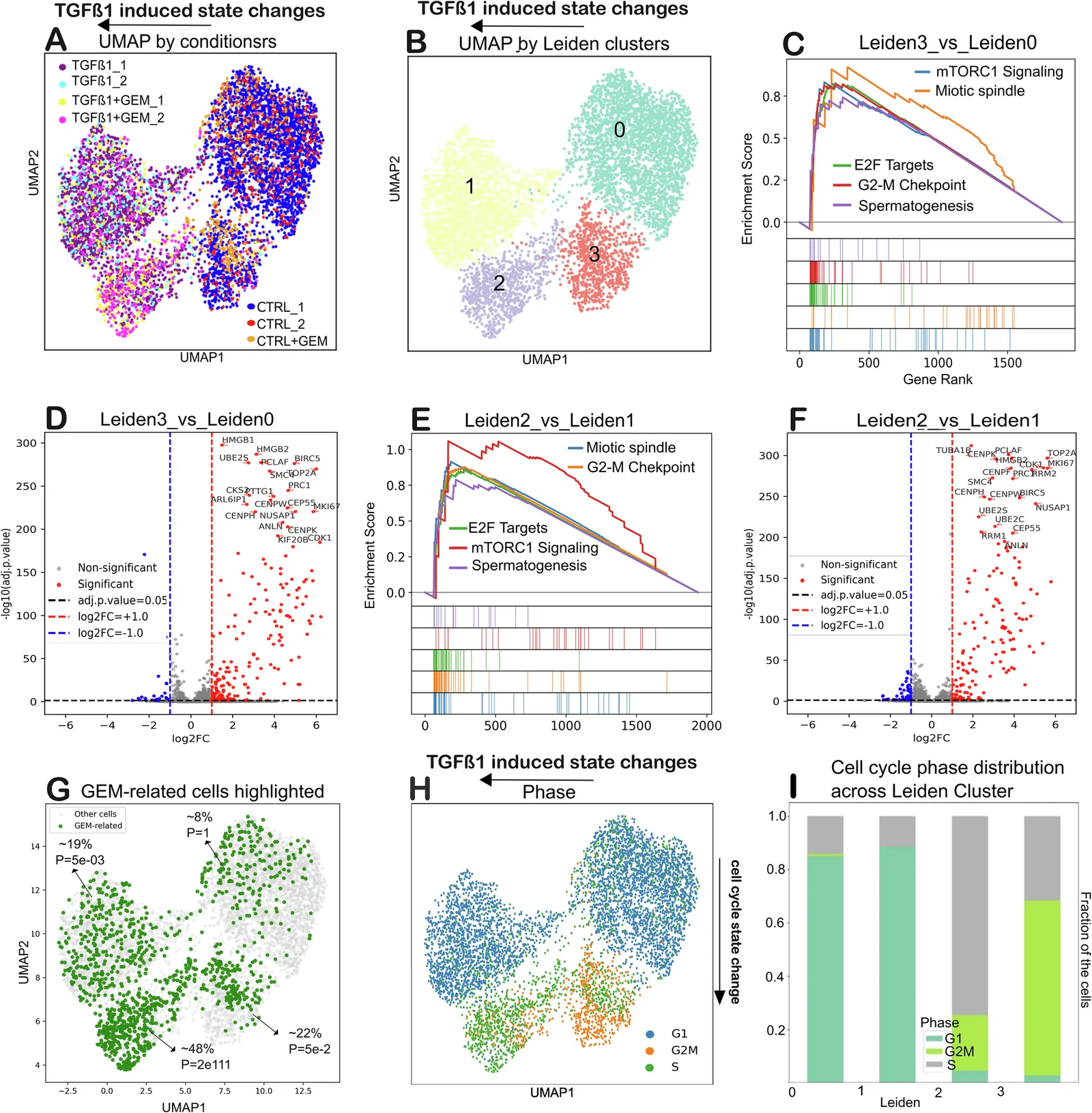
Single-cell clustering of 3D PANC-1 tissues identifies four cell states defined by cell-cycle phase and TGF-β1 status. **A** UMAP projection of scRNA-seq data from all four conditions (CTRL, CTRL + GEM, CTRL + TGF-β1, CTRL + GEM + TGF-β1), colored by condition. Biological replicates co-localise as expected; CTRL + GEM lacks replicates (only surviving cells captured). **B** Unsupervised Leiden clustering identifies four stable subpopulations. Clusters 0 and 1 are predominantly G1-phase; clusters 2 and 3 are enriched for S and G2/M phase cells. **C** GSEA comparing Leiden cluster 3 versus cluster 0 (CTRL and CTRL + GEM conditions), showing enrichment of mitotic spindle, E2F target, and G2/M checkpoint pathways in cluster 3. **D** Volcano plot of differential gene expression between Leiden cluster 3 and cluster 0. Top upregulated genes include RRM2, CDK1, BIRC5, NUSAP1, MKI67, and TK1. **E** GSEA comparing Leiden cluster 2 versus cluster 1 (CTRL + TGF-β1 and CTRL + GEM + TGF-β1 conditions), revealing similar cell cycle checkpoint enrichment under TGF-β1 stimulation. **F** Volcano plot of differential gene expression between Leiden cluster 2 and cluster 1. **G** GEM-surviving cells (green) projected onto the UMAP. Survivors distribute across pre-existing clusters rather than forming a distinct population. Cluster enrichment percentages and Fisher’s exact test *p*-values are annotated: cluster 2 shows the strongest enrichment (48%, *p* ~ 2×10^−111^). **H** UMAP colored by predicted cell-cycle phase (G1, S, G2/M) across all conditions, showing enrichment of cycling cells in clusters 2 and 3. **I** Bar chart showing the distribution of predicted cell-cycle phases across the four Leiden clusters, quantifying the phase-specific enrichment summarised in panel H.

**Fig. 3:**
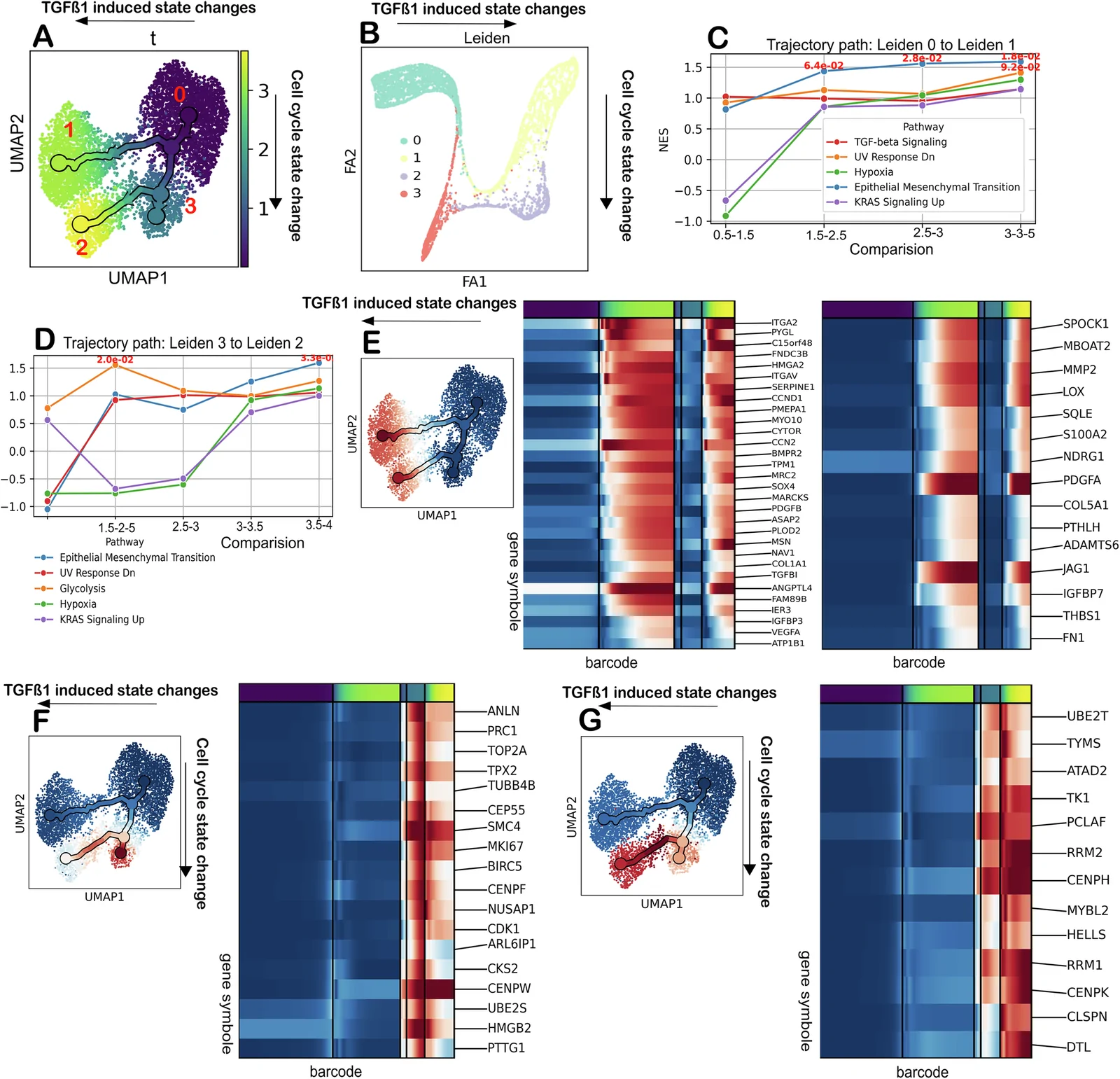
Pseudotime trajectory inference resolves continuous TGF-β1-induced state transitions and identifies GEM-associated gene programs in 3D PANC-1 tissues. **A** UMAP projection colored by pseudotime (violet: early [0], yellow: late [3.7]), with Leiden cluster identities annotated in red. Two parallel trajectories originate from G1-phase control subpopulations (clusters 0 and 3) and progress to TGF-β1-associated states (clusters 1 and 2) with increasing pseudotime. **B** Force Atlas 2 (FA2) graph layout representation colored by Leiden cluster, illustrating the continuity of the transcriptional landscape between clusters. **C**, **D** Trajectory-resolved GSEA (MSigDB Hallmark gene sets) showing normalized enrichment scores (NES) across binned pseudotime along the two parallel trajectories: cluster 0 → 1 (**C**) and cluster 3 → 2 (**D**). Bins represent pseudotime intervals of 0.5; only bins with ≥50 cells are shown. Pathways shown are the top-ranked MSigDB Hallmark gene sets by NES reaching significance (FDR < 0.25) in at least one pseudotime bin. **E**–**G** Heatmaps of fitted expression along pseudotime (columns, ordered by trajectory) for three co-regulated gene modules; colour indicates per-gene (row-wise) z-scored fitted expression (red, above the gene’s mean along pseudotime; blue, below), rescaled to improve within-module contrast. **E** TGF-β1-induced genes, ordered by temporal onset. **F** Cell cycle–associated genes changing during G1 → S progression. **G** Genes with overlapping TGF-β1 and cell cycle expression, enriched in GEM-surviving cells. Trajectory color bars denote pseudotime progression (0–3.7).

**Fig. 4:**
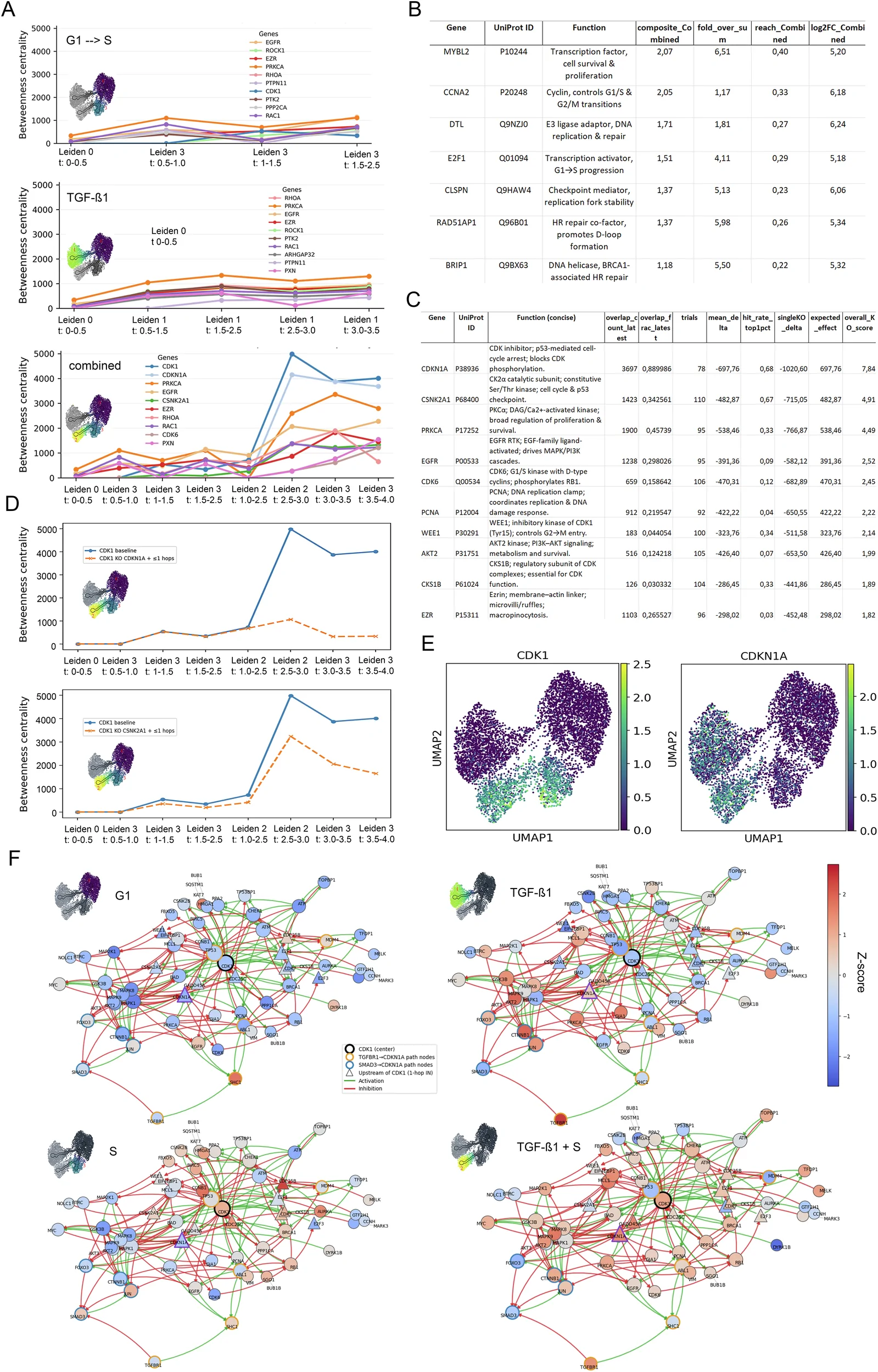
Network determinants of the Combined (G1 → S + TGF-β1) state. **A** Betweenness centrality of network nodes across pseudotime along the G1 → S, TGF-β1, and Combined trajectories. Leiden clusters are ordered on the x-axis by pseudotime position rather than cluster index (Leiden 2 lies between Leiden 3 and Leiden 1, reflecting its intermediate state in the combined trajectory). **B** Genes with high network-mediated influence in the Combined state, passing the synergy filter (composite_Combined > composite_TGFB + composite_CC; reach_Combined > reach_TGFB + reach_CC; log2FC_Combined > 0). The composite score is (Personalized PageRank reach) × |log₂FC| on the undirected PPI (α = 0.85; top-perturbed *N* = 20); fold_over_sum = composite_Combined / (composite_TGFB + composite_CC), where values > 1 indicate synergy. See Methods for full definitions. **C** Upstream nodes ranked by co-occurrence on CDK1-mediated shortest paths (overlap_count/frac_latest) and by the reduction in CDK1 betweenness upon their removal in randomized knockout simulations (mean ΔCDK1 betweenness, hit_rate_top1pct, singleKO_delta); overall_KO_score integrates these signals (weights: 0.35, 0.35, 0.20, 0.10, respectively). **D** CDK1 betweenness across pseudotime (G1 → S and TGF-β1 trajectories) before and after knockout of CDKN1A and CSNK2A1 with their direct downstream interactors. **E** Expression of CDK1 and CDKN1A on the UMAP. **F** CDK1-anchored network across four states (G1, S, TGF-β1, S + TGF-β1). Edges: green = activating, red = inhibitory, grey dashed = other; arrows indicate direction. Node colour encodes the expression z-score (diverging scale, globally normalized across panels). CDK1 is outlined with a thicker border. CDK1 1-hop downstream targets in the latest Combined bin are shown in Supplementary Fig. 8.

Building on companion bulk analysis of this dataset ^14^, we applied scRNA-seq across four conditions (control, GEM, TGF-β1, GEM + TGF-β1) to resolve individual cell states and their transitions (Fig. 2). Pseudotime trajectory inference reconstructed continuous routes of cell-state change, including G₁ → S progression, TGF-β1-induced EMT, and their intersection (Fig. 3). We then tracked how protein–protein interaction network topology shifts along these trajectories rather than comparing static snapshots, to identify regulatory bottlenecks emerging specifically from the co-occurrence of transcriptional programs (Fig. 4). Centrality analysis revealed a sharp, synergistic spike in CDK1 and CDKN1A betweenness centrality exclusively in the combined G₁ → S + TGF-β1 state, absent in either program alone. In silico knockout simulations identified upstream regulators whose removal most effectively unloads this bottleneck, generating experimentally testable hypotheses for therapeutic interference.

### Single-cell clustering identifies four cell states defined by cell-cycle phase and TGF-β1 status

UMAP projections segregated the four experimental conditions into distinct regions, with biological replicates co-localising as expected (Fig. 2A). Leiden clustering yielded four stable subpopulations (Fig. 2B). Because PANC-1 is a monoclonal pancreatic cancer cell line, conventional cell type annotation (e.g. epithelial, stromal, immune) is not applicable—all cells share the same identity. Leiden clusters therefore represent distinct cell-cycle and phenotypic attractor states rather than distinct cell types, and are labelled numerically accordingly. Clusters 0 and 1 were predominantly G1-phase; clusters 2 and 3 were enriched for S and G2/M phase cells, with elevated CDK1, AURKA, E2F targets, and G2/M checkpoint programs (Fig. 2H–I; Supplementary Fig. 4). Note that the CTRL + GEM condition lacks replicates, as only surviving cells were captured; this group should be interpreted accordingly.

Within control PANC-1 cells, clusters 3 versus 0 differed substantially in cell cycle regulation: ~200 genes were upregulated including RRM2, CDK1, BIRC5, NUSAP1, CENPW, MKI67, UBE2C, UBE2S, and TK1, with marked enrichment of mitotic spindle, E2F target, and G2/M checkpoint pathways (Fig. 2C–D; Supplementary Fig. 2A). TGF-β1-stimulated cells showed analogous inter-cluster heterogeneity (clusters 2 vs. 1): ~148 genes upregulated with similar mitotic checkpoint enrichment (Fig. 2E–F; Supplementary Fig. 2C). The full TGF-β1-induced gene expression landscape (clusters 1 vs. 0; ~800 differentially expressed genes) is provided in Supplementary Fig. 2D and warrants the systematic trajectory analysis that follows.

GEM-surviving cells did not form a distinct cluster but distributed across pre-existing Leiden clusters (Fig. 2G). The strongest enrichment was in cluster 2 (48%, Fisher’s exact *p* ~ 2×10⁻¹¹¹), the S-phase + EMT population, followed by cluster 3 (22%, *p* ~ 0.05) and cluster 1 (20%, *p* ~ 5×10⁻³), with cluster 0 showing no enrichment. GEM survivors shared a common gene expression signature involving H2AZ1, CENPW, TK1, RRM1, HELLS, CLSPN, RRM2, MYBL2, and NUSAP1, genes linked to replication stress tolerance and kinetochore regulation (Supplementary Fig. 3). Ki-67 immunostaining confirmed that proliferative cells persist after GEM exposure, with the combined TGF-β1 + GEM condition showing the strongest enrichment (~55% Ki-67 positive; Fig. 1C).

These findings establish that GEM resistance does not arise as a discrete new transcriptional state but reflects selective enrichment within pre-existing proliferative S-phase and G2/M subpopulations. The complexity of TGF-β1-induced expression changes (~600–800 differentially expressed genes per comparison) and the heterogeneous distribution of GEM survivors across clusters motivate a trajectory-based analysis to resolve the continuous regulatory dynamics underlying chemoresistance.

### Pseudotime trajectory inference resolves continuous TGF-β1-induced state transitions and identifies GEM-associated gene programs

Pseudotime trajectory inference ordered cells along continuous transcriptional routes originating from Leiden cluster 0 (low E2F targets, low mitotic activity) and progressing toward TGF-β1-associated states (Fig. 3A–B; Supplementary Fig. 5). Two parallel trajectories were identified. One from cluster 0 → 1 and one from cluster 3 → 2, reflecting the dual substructure observed in static clustering.

Trajectory-resolved GSEA revealed the pathway dynamics driving these transitions (Fig. 3C–D). Along both trajectories, TGF-β1 signalling and EMT were persistently and strongly upregulated. Hypoxia, KRAS signalling, and glycolysis were also elevated, though with less consistent significance. Epithelial identity genes (KRT8, KRT18, KRT19, CAV1) were progressively downregulated (Supplementary Fig. 6), consistent with coordinated EMT progression.

Gene expression clustering along the trajectories resolved three temporally distinct programs with independent clinical validation (Fig. 3E–G; Supplementary Fig. 7). The first group (Fig. 3E) comprises genes sequentially induced by TGF-β1: early-response genes (SERPINE1, MYO10, TPM1, HMGA2, VEGFA) are activated first, followed by a later wave (COL1A1, SPOCK1, MBOAT2, LOX), and a final late-onset set (ADAMTS6, COL5A1, THBS1, FN1, S100A2, PTHLH). Representative genes from this group, MBOAT2, S100A2, and FN1, show significant association with poor survival in PDAC patients (HR ~ 1.44, 1.65, and 1.50, respectively; Kaplan–Meier Plotter; Supplementary Fig. 7A). The second group (Fig. 3F) reflects cell cycle–associated changes during G1 → S progression, with expression peaking in clusters 2 and 3. Key genes TPX2, TOP2A, and TK1 previously linked to GEM resistance carry HRs of ~1.41, 1.43, and 1.30 (Supplementary Fig. 7B). The third group (Fig. 3G) exhibits overlapping expression from both TGF-β1 induction and cell cycle programs and is notably enriched in GEM-surviving cells: ~48% of GEM survivors localise to the combined S-phase + EMT cluster 2, ~22% to the cell cycle cluster 3, and ~19% to the TGF-β1 cluster 1. Genes in this group, HELLS, MYBL2, PLAU, and DTL, carry HRs of ~1.33–1.47, consistent with roles in chemoresistance and tumour persistence (Supplementary Fig. 7C).

The clinical concordance across all three gene groups confirms that the transcriptional programs resolved by trajectory analysis in the 3D tissue model reflect biology relevant to patient outcomes. GEM resistance does not arise from a distinct new transcriptional state but from selective enrichment within cells co-activating both proliferative and EMT programs simultaneously, a regulatory overlap examined mechanistically in the following network analysis.

### Trajectory-coupled network topology reveals an emergent CDK1–CDKN1A bottleneck in the combined S-phase** + **TGF-β1 state

To determine how regulatory network control shifts under drug pressure, we tracked betweenness centrality, a measure of how many shortest communication paths traverse each node, across pseudotime bins along three trajectories: G1 → S, TGF-β1, and their combined state (Fig. 4A). Along the G1 → S trajectory, classic cell-cycle nodes rose in centrality (CDK1, EGFR, PRKCA). Under TGF-β1 alone, EMT and cytoskeletal regulators gained modest centrality (RHOA, ROCK1, EZR). Strikingly, in the combined S-phase + TGF-β1 condition, CDK1 and CDKN1A/p21 showed an exceptional synergistic spike absent in either trajectory alone (Δbetweenness ≈ 5,000 and ≈ 4,000 respectively; Fig. 4A). This spike arises because CDK1 and CDKN1A become the primary bridge connecting two otherwise independent modules, the S-phase/mitotic program and the EMT/TGF-β1/SMAD program, when both programs are simultaneously active. Consistent with this bridging role, when betweenness is computed across the entire reconstructed network, CDK1 and CDKN1A rank among the highest-centrality hubs of all 2588 nodes (rank 4 and 14, respectively), well separated from the heavy-tailed bulk of low-centrality nodes (Supplementary Fig. 9). The enrichment of GEM survivors in this combined state (cluster 2, 48%) directly links this topological bottleneck to chemoresistance.

To identify which genes amplify this bottleneck, we ranked nodes in the combined state by a composite influence score (Personalized PageRank reach × |log₂FC | ) with a synergy filter requiring scores to exceed the sum of single-condition scores (Fig. 4B). Three functional groups converged on the CDK1/CDKN1A gate. On the pro-mitotic side, MYBL2 and E2F1 showed the strongest synergy (fold-over-sum ≈ 6.5 and 4.1) and broad network reach (≈0.40 and 0.29), driving S-phase and mitotic target expression^39^. CCNA2 directly activates CDK1 complexes. On the restraint side, CLSPN (ATR → CHK1 mediator; synergy ≈ 5.1) and DTL (CRL4-CDT2 ligase; synergy ≈ 1.81) load the gate from the checkpoint arm, suppressing CDK1 activation via CHK1-mediated CDC25 inhibition and modulating the p21/CDT1 axis. Supporting both sides, RAD51AP1 and BRIP1 (synergy ≈ 6.0 and 5.5) provide homologous recombination and Fanconi pathway support, stabilising S-phase under genotoxic stress. Together, these three modules define a state where pro-mitotic drive and checkpoint restraint converge simultaneously on CDK1/p21, a configuration that sustains S-phase without completing mitosis (schematised in Supplementary Fig. 10).

To identify which upstream regulators sustain the CDK1 bottleneck and could be targeted to disrupt it, we combined shortest-path overlap analysis with randomised knockout (KO) simulations across trajectory subnetworks (Fig. 4C–D). Two candidates dominated: CDKN1A/p21 and CSNK2A1/CK2α. CDKN1A co-occurs on ~89% of CDK1-mediated shortest paths in the latest combined bin; its removal reduces CDK1 betweenness by ~1,021 (exact KO), and this effect is highly robust (top-1% hit-rate 0.68; overall KO score 7.84). CSNK2A1 shows a similar but smaller profile (34% path overlap; KO Δ ≈ −715; KO score 4.91). Expression analysis confirms the mechanistic logic: CDK1 is induced by S-phase progression, while CDKN1A is driven by TGF-β1/SMAD3 rather than cell-cycle entry (Fig. 4E). Their co-occurrence, S-phase pushing CDK1 up while TGF-β1 maintains p21, creates the narrow isthmus where the two programs converge. CDK1 downstream targets in the latest combined bin, including direct 1-hop targets MCL1, EIF4EBP1, RRM2, RUNX1, and TK1, are provided in Supplementary Fig. 8.

Network visualisation across four states (G1, S, TGF-β1, S + TGF-β1) confirms this interpretation (Fig. 4F): CDK1 acquires extensive cross-module connections exclusively in the combined state, with activating edges from the mitotic arm (MYBL2, CCNA2) and inhibitory edges from the checkpoint arm (CDKN1A, WEE1) converging simultaneously—a topology absent in either single program, and summarised as a molecular-gate schematic in Supplementary Fig. 10.

### The CDK1–CDKN1A–WEE1 bottleneck is enriched in metastatic PDAC and predicts disease progression in a 726,107-cell patient atlas

To determine whether the CDK1–CDKN1A–WEE1 bottleneck identified in our 3D tissue model reflects human tumour biology, we cross-examined key signatures in a comprehensive PDAC single-cell atlas (726,107 cells, 231 patients, 12 studies; Loveless et al.^15^). From the full atlas, we extracted 300,577 ductal and cycling ductal cells and computed PHATE embeddings to preserve continuous trajectory structure. Cells were scored for the four Leiden-derived signatures from the tissue model: L0 (epithelial baseline), L1 (TGF-β/EMT), L2 (S-phase + EMT bottleneck), and L3 (G2/M proliferative). Multi-level mapping confirmed that L2 (bottleneck) cells localise specifically at the junction between EMT and proliferative branches in both the full-atlas UMAP and the epithelial PHATE embedding, with triple-positive (CDK1⁺/CDKN1A⁺/WEE1⁺) cells concentrated at this interface (*n* = 2224; Supplementary Fig. 11A–I). Metastatic cells are enriched at the proliferative branch tips (Supplementary Fig. 11E); gemcitabine survivors are predominantly G1-arrested (73%), while FOLFIRINOX survivors retain more cycling cells in the bottleneck region (Supplementary Fig. 11J–L).

**Fig. 5:**
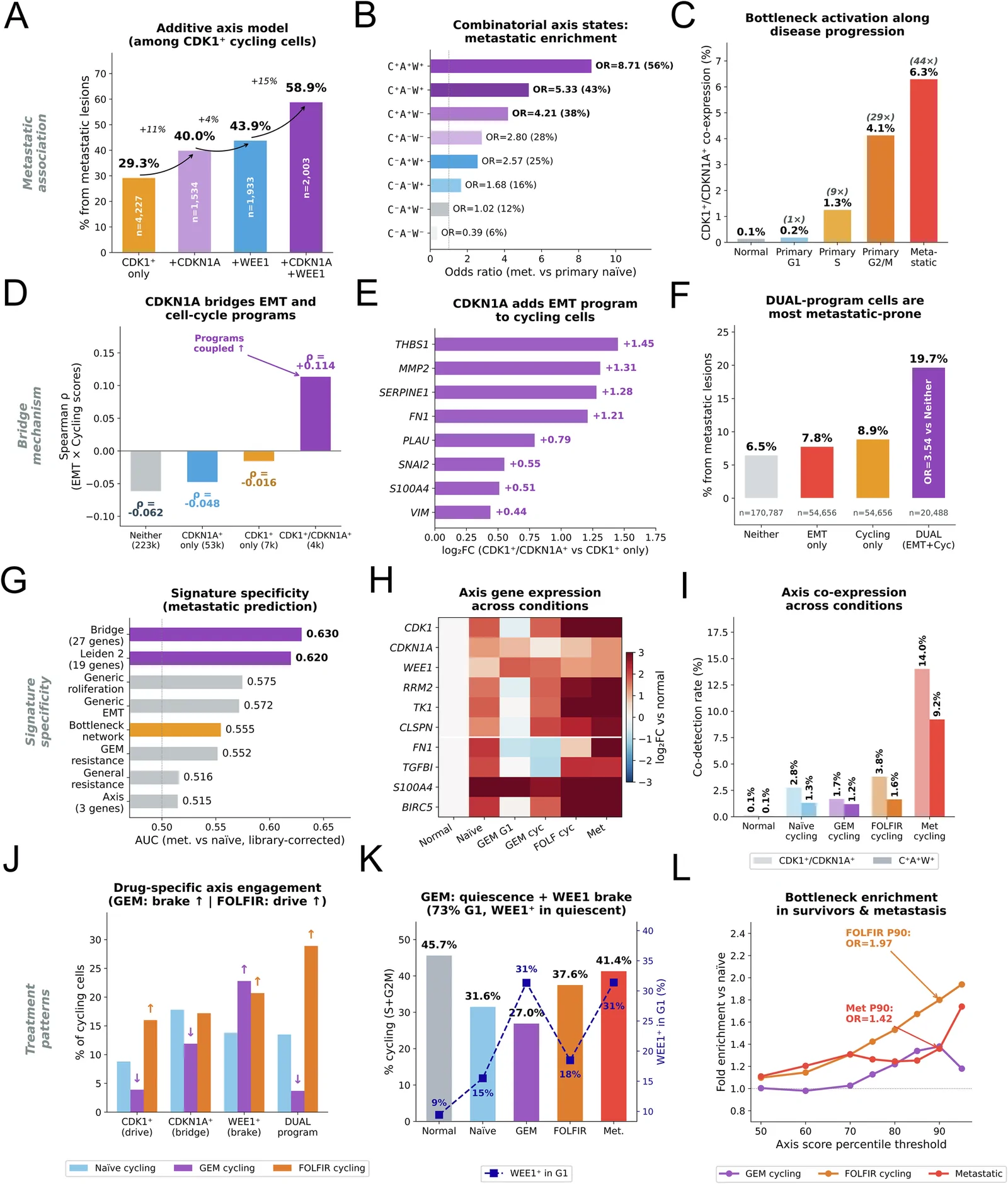
Quantitative validation of the CDK1–CDKN1A–WEE1 bottleneck axis: metastatic association, bridge mechanism, and treatment-specific patterns. **A**–**C** Metastatic association. **A** Additive axis model: among CDK1^+^ cycling cells (*n*  =  9697), the fraction originating from metastatic lesions increases with each additional axis component (CDK1^+^ only: 29.3%; +CDKN1A: 40.0%; +WEE1: 43.9%; +CDKN1A + WEE1: 58.9%). **B** Odds ratios for metastatic enrichment across all eight combinatorial states of CDK1/CDKN1A/WEE1 expression (Fisher’s exact test versus treatment-naïve primary; OR and percent metastatic shown for each state). Triple-positive cells show OR  =  8.71 (56% metastatic); triple-negative cells are protective (OR  =  0.39, 6.5% metastatic). **C** CDK1^+^/CDKN1A^+^ co-expression rate along disease progression, from normal epithelium (0.1%) through primary tumor cell-cycle phases (G1: 0.2%; S: 1.3%; G2/M: 4.1%) to metastatic cells (6.3%). Fold enrichment over normal is annotated above each bar. **D**–**F** Bridge mechanism. **D** Spearman correlation between aggregate EMT and cell-cycle program scores, stratified by CDK1/CDKN1A expression state. In non-expressing cells, programs are anti-correlated (*ρ*  =   − 0.062); in CDK1^+^/CDKN1A^+^ cells, the correlation reverses to positive (*ρ*  =   + 0.114, p  =  5.1 × 10^−13^), demonstrating that co-expression specifically couples the two programs. **E** Log_2_ fold change of EMT genes in CDK1^+^/CDKN1A^+^ versus CDK1^+^-only cycling cells, showing that CDKN1A co-expression adds an EMT transcriptional program to actively cycling cells. **F** Fraction of cells from metastatic lesions across four program categories: neither EMT nor cycling (6.5%), EMT only (7.8%), cycling only (8.9%), and DUAL-program cells activating both (19.7%; OR  =  3.54 versus neither). **G**–**I** Signature specificity. **G** AUC for discriminating metastatic versus treatment-naïve cells, corrected for library size, across multiple gene signatures. Bridge score (27 genes, AUC = 0.630) and Leiden 2 (19 genes, AUC = 0.620) outperform generic proliferation (0.575), generic EMT (0.572), established resistance panels (0.515–0.555), and the 50-gene bottleneck network score (AUC = 0.535, orange bar). **H** Heatmap of log₂ fold change for axis and associated genes across conditions relative to normal epithelium. Cell-cycle genes (top; CDK1, CDKN1A, WEE1, RRM2, TK1, CLSPN) are separated from EMT genes (bottom; FN1, TGFBI, S100A4) by a white line. GEM G1-arrested survivors show selective WEE1 upregulation without CDK1 elevation; FOLFIRINOX cycling survivors show broad upregulation of both gene sets. **I** CDK1^+^/CDKN1A^+^ co-detection (light bars) and triple-positive C^+^A^+^W^+^ co-detection (dark bars) rates across conditions, showing progressive enrichment from normal (0.1%) to metastatic cycling cells (14.0% and 9.2%, respectively). **J**–**L** Treatment-specific patterns. **J** Prevalence of each axis component among cycling cells from treatment-naïve (blue), gemcitabine (purple), and FOLFIRINOX (orange) groups. Arrows indicate significant enrichment (↑) or depletion (↓) relative to naïve. Gemcitabine depletes CDK1^+^ (OR  =  0.42) and DUAL-program cells (OR  =  0.25), but enriches WEE1^+^ (OR  =  1.84); FOLFIRINOX enriches all components. **K** Fraction of cycling cells (S + G2/M) by treatment group (bars), with WEE1^+^ detection rate among G1-arrested cells overlaid (blue dashed line, right axis). Gemcitabine survivors are most quiescent (27.0%) with highest WEE1 G1-detection (31%), consistent with WEE1-mediated checkpoint arrest. **L** Fold enrichment over treatment-naïve cells at increasing axis-score percentile thresholds for gemcitabine cycling survivors (purple), FOLFIRINOX cycling survivors (orange), and metastatic cells (red). Progressive enrichment at higher thresholds confirms dose-dependent bottleneck engagement.

Additive metastatic enrichment of the three-component axis. Among CDK1⁺ cycling cells (n = 9,697), the proportion from metastatic lesions increased with each additional axis component: CDK1⁺ alone 29.3%; with CDKN1A 40.0%; with WEE1 43.9%; with both 58.9% (Fig. 5A). Systematic analysis of all eight combinatorial states confirmed a clear hierarchy: the triple-positive state (C + A + W + ) showed the strongest metastatic association (OR = 8.71, 56.5% metastatic; Fisher’s exact test versus treatment-naïve primary), while the triple-negative state was protective (OR = 0.39; Fig. 5B). CDK1⁺/CDKN1A⁺ co-expression increased monotonically along disease progression from 0.1% in normal epithelium to 6.3% in metastatic cells (44-fold enrichment; Fig. 5C), paralleling the trajectory from quiescence through cell-cycle entry to invasive capacity.

CDKN1A functionally couples EMT and cell-cycle programs. A central prediction of the bottleneck model is that CDKN1A does not merely co-occur with CDK1 but actively bridges the two transcriptional modules. We tested this by computing Spearman correlations between aggregate EMT and cell-cycle program scores across four mutually exclusive expression states (Fig. 5D). In cells expressing neither gene (*n* = 223,000), EMT and cell-cycle scores were anti-correlated (*ρ* = −0.062), reflecting established program antagonism. Strikingly, in CDK1⁺/CDKN1A⁺ co-expressing cells, this correlation reversed to positive (*ρ* = +0.114, *p* = 5.1×10⁻¹³)—CDKN1A co-expression specifically enables simultaneous activation of both programs. Consistent with this, CDK1⁺/CDKN1A⁺ cycling cells showed marked upregulation of EMT effectors relative to CDK1⁺-only cells: THBS1 (+1.45 log₂FC), MMP2 (+1.31), SERPINE1 (+1.28), FN1 (+1.21; all *p* < 10⁻⁷⁶; Fig. 5E). DUAL-program cells (above 75th percentile for both L1 and L3) were the most metastatic-prone population (19.7% metastatic versus 6.5% in neither program; OR = 3.54; Fig. 5F), and CDK1⁺/CDKN1A⁺ cells were 8.62-fold enriched for DUAL-program membership.

Bottleneck signatures outperform generic gene sets. The 27-gene bridge signature achieved AUC = 0.630 and the 19-gene Leiden 2 signature AUC = 0.620 for discriminating metastatic from treatment-naïve cells—substantially outperforming generic proliferation (0.575), generic EMT (0.572), and established GEM resistance panels (0.552; Fig. 5G). The three-gene axis binary co-detection (OR = 8.71) outperformed all continuous scores. Gene-level expression confirmed co-elevation of cell-cycle (CDK1, RRM2, TK1, CLSPN) and EMT genes (FN1, TGFBI, S100A4) in metastatic cells, while GEM G1-arrested survivors showed selective WEE1 upregulation without CDK1 elevation, consistent with checkpoint-mediated arrest (Fig. 5H). CDK1⁺/CDKN1A⁺ co-detection was highest in metastatic cycling cells (14.0%) and FOLFIRINOX survivors (3.8%), compared with 2.8% in naïve cycling cells and 0.1% in normal epithelium; triple-positive co-detection followed the same hierarchy (Fig. 5I).

Drug-specific survival strategies. Gemcitabine survivors were depleted for CDK1⁺ cells (OR = 0.42) and DUAL-program cells (OR = 0.25) but enriched for WEE1⁺ (OR = 1.84), with G1-arrested survivors showing 31% WEE1 detection versus 16% in naïve G1—consistent with WEE1-mediated checkpoint arrest as the dominant GEM survival mechanism (Fig. 5J). FOLFIRINOX survivors showed the opposite: enrichment for CDK1⁺ (OR = 1.96), DUAL program (OR = 2.61), and WEE1⁺ (OR = 1.63), consistent with continued cell-cycle transit through the bottleneck. The quiescence pattern confirmed this interpretation: gemcitabine survivors were the most quiescent population (27.0% cycling versus 31.6% naïve), while metastatic cells showed the highest proliferative capacity (41.4% cycling; Fig. 5K). Axis score percentile enrichment confirmed dose-dependent bottleneck engagement: at the 90th percentile, FOLFIRINOX survivors showed 1.97-fold enrichment over naïve cells, metastatic cells 1.42-fold (Fig. 5L).

We note that each treatment arm derives from a single study with limited patient numbers and no matched pre-treatment samples; treatment-specific patterns are therefore observational and require validation in matched cohorts.

## Discussion

This study integrates three complementary advances to resolve the regulatory architecture of gemcitabine resistance in PDAC: a physiologically relevant 3D tissue platform enabling near-clinical drug responses, a systems biology framework tracking how PPI network topology continuously shifts across pseudotime trajectories rather than comparing static snapshots, and cross-examination in a 726,107-cell patient atlas that simultaneously validates both the model and the method. Together, these identify the CDK1–CDKN1A–WEE1 bottleneck as a recurrent feature of human PDAC biology associated with metastatic propensity and drug-specific survival strategies.

Conventional 2D PDAC cultures have artificially high proliferation indices that mask the selective dynamics underlying chemoresistance^16^. Our SISmuc-based 3D model reaches a homeostatic proliferation index of ~35% Ki-67 positive after 11 days, closely matching the clinical mean of ~26% in PDAC patients^11–13^, and enables tissue-niche-specific GEM responses including preferential eradication of crypt-localised cells. Critically, GEM treatment in our model increases Ki-67 positivity rather than reducing it, a counterintuitive finding that reflects genuine selection of a resistant proliferative subpopulation rather than uniform cytotoxicity. This observation is consistent with Striefler^11^, who found that GEM-treated PDAC tumours with lower Ki-67 indices showed longer progression-free and overall survival—implying that Ki-67-high survivors represent the clinically dangerous residual population. The single-cell resolution of scRNA-seq applied to this platform resolves which specific cell states within the 3D tissue survive treatment, a granularity impossible in bulk or 2D approaches.

GEM-surviving cells concentrate in the S and G2/M-enriched Leiden clusters, characterised by high CDK1, BIRC5, TOP2A, TPX2, AURKA, and RRM2 expression. This is consistent with GEM’s established mechanism: by disrupting DNA synthesis and inhibiting ribonucleotide reductase, GEM induces S-phase arrest and activates ATR/CHK1 signalling, eliminating sensitive cells while selecting for those capable of sustaining replication under stress^17,18^. The AURKA/TPX2 axis shortens S-phase duration and bypasses arrest^19–21^; RRM2 replenishes nucleotide pools^22–24^; BIRC5 and mTORC1 promote survival^25–28^; and CDK1 dysregulation enables G2/M checkpoint bypass^29^. These mechanisms are well-established in 2D models but our 3D platform allows their observation in a tissue-niche context where microenvironmental heterogeneity, absent in 2D, modulates which cells access these survival programs.

TGF-β1 introduced a distinct attractor state marked by progressive induction of FN1, THBS1, COL5A1, SPOCK1, and MBOAT2 with concurrent loss of KRT18 and KRT19, consistent with canonical and non-canonical EMT signalling through SMAD, PI3K/AKT, KRAS/MAPK, and mTOR^30^. Several late-induced genes carry strong prognostic weight: S100A2 and FN1 are established GEM resistance markers^31–34^; MBOAT2 is an unfavourable biomarker^35^. TGF-β1 overrides inherent cell-cycle attractors, reorienting cells toward an EMT phenotype that can simultaneously arrest the cell cycle and promote motility^36,37^—explaining independent enrichment of GEM survivors in the TGF-β1 cluster. Clinical trials targeting TGF-β1^38^ or EMT alone have shown modest benefits in PDAC, consistent with the view that neither program alone is the primary target, their co-occurrence is.

Existing PPI network analyses of PDAC have typically compared expression-weighted networks across discrete conditions, capturing which pathways are active at a given state but missing how network control architecture shifts as cells transition between states. By contrast, tracking betweenness centrality continuously across pseudotime bins revealed an emergent bottleneck invisible in any single comparison: CDK1 and CDKN1A show a sharp synergistic centrality spike (Δbetweenness ≈ 5000 and 4000, respectively) exclusively when G1 → S progression and TGF-β1-induced EMT co-occur (absent in either trajectory alone). This spike arises because the two programs activate orthogonal regulatory arms that converge on a single gate: S-phase progression drives CDK1 via E2F1/MYBL2/CCNA2^39^, while TGF-β1/SMAD3 independently induces p21 in a p53-independent manner^40^, directly inhibiting CDK1 and blocking S → M transition. The resulting S-phase persistence state—cells primed for division yet held before mitosis—is sustained by replication fork protection (RAD51AP1, BRIP1), nucleotide supply (RRM2), and checkpoint stabilisation (CLSPN → CHK1). This aligns with evidence that EMT programs enhance replication-stress tolerance and activate DDR^41,42^, but the dynamic network framework uniquely identifies *when* and *why* CDK1/p21 becomes the critical control point rather than just noting their co-expression.

The PDAC atlas analysis^15^ (726,107 cells, 231 patients, 12 studies spanning the full disease spectrum from normal epithelium to metastasis) provides a stringent test: concordance between signatures derived from a single cell line under four defined conditions and patterns across 231 patients in 12 independent studies simultaneously validates the 3D tissue model as capturing clinically relevant biology and the trajectory-coupled network framework as extracting generalisable mechanistic insight. This joint validation is rarely achievable in single-cell cancer studies. Triple-positive cells localise specifically at the EMT–proliferative branch junction rather than distributing randomly (OR = 8.71 for metastatic origin), exceeding the discriminative power of generic proliferation, EMT, and established drug-resistance gene panels. The correlation-reversal finding, EMT–cell-cycle anti-correlation (*ρ* = −0.062) reversing to positive coupling (*ρ* = +0.114) specifically in CDK1⁺/CDKN1A⁺ cells, provides direct molecular confirmation of the bridging function predicted by the network model. These observations are independently supported by Werba^43^, who found chemotherapy-treated PDAC cells enriched for EMT programs relative to treatment-naïve tumours, consistent with chemotherapy selecting for cells engaging the EMT–cell-cycle bottleneck. Treatment data further distinguish two survival strategies: GEM survivors adopt a WEE1-dominant checkpoint arrest pattern (depleted CDK1⁺, enriched WEE1⁺), while FOLFIRINOX survivors maintain active cycling through the full bottleneck—a distinction with direct therapeutic implications.

The bottleneck topology identifies the ATR–CHK1–WEE1 axis as the most direct therapeutic vulnerability, since inhibiting these kinases collapses S-phase persistence by forcing premature mitotic entry^44,45^. Combination regimens are already standard in oncology, and WEE1, CHK1 and ATR inhibitors are in trials with gemcitabine^46,47^; our findings therefore do not propose combination per se, but refine its rationale. Rather than combining agents to raise the general barrier to escape, the emergent bottleneck defines a state-dependent vulnerability rather than a static target, present only where the G1 → S and TGF-β1 programs converge—the chemo-tolerant subpopulation—and absent from the bulk tumour. The therapeutic goal thus becomes selective collapse of a topological control point rather than uniform cytotoxic intensification, which shifts the emphasis from which pathways to co-target toward when and in which cells the bottleneck is engaged, and motivates therapy that is timed and stratified to the S-phase-persistence state. Clinical translation is already active: adavosertib with gemcitabine and radiation achieved 21.7 months median overall survival in locally advanced PDAC^46^, and SRA737 with gemcitabine showed favourable tolerability^47^. Our atlas-derived survival patterns suggest how such regimens might be matched to patients. GEM survivors with a WEE1-dominant pattern may benefit most from WEE1 inhibition, whereas FOLFIRINOX survivors engaging the full DUAL program may require combined ATR–CHK1–WEE1 targeting; upstream, CDK4/6 inhibition can dampen the E2F/MYBL2 program, although compensatory TGF-β/SMAD-dependent EMT induction limits monotherapy efficacy^48^. The CDK1–CDKN1A–WEE1 composite signature score could accordingly serve as a candidate companion diagnostic, prioritising patients with high CDK1⁺/WEE1⁺ co-detection and a low proliferating fraction for checkpoint inhibitor trials, and those with full DUAL-program engagement for combined regimens. The convergent principle that durable PDAC responses require simultaneous disruption of multiple signalling nodes is supported by preclinical combination studies^48,49^ and underscores the need for biomarker-driven patient selection in future trials.

The model uses a single PANC-1 line; validation in patient-derived organoids is required to assess generalisability across mutational backgrounds. Pseudotime is inferred rather than measured, and network topology depends on OmniPath curation completeness. Treatment comparisons in the atlas are observational; each arm derives from a single study without matched pre-treatment samples.

We establish a physiologically relevant 3D PDAC tissue platform enabling single-cell mapping of drug-induced resistance trajectories, and introduce a systems biology framework, pseudotime trajectory inference integrated with dynamic PPI network topology analysis, that resolves emergent regulatory bottlenecks invisible to static approaches. Cross-examination of tissue model findings in a 726,107-cell patient atlas supports both the experimental platform and the analytical framework simultaneously: signatures identified in a controlled 3D system under four defined conditions recapitulate the most aggressive biology in human PDAC at population scale. The CDK1–CDKN1A–WEE1 axis defines an S-phase persistence state where proliferative drive and TGF-β1-induced restraint converge, generating a chemo-tolerant subpopulation likely underlying relapse and minimal residual disease. Triple-positive cells show 8.7-fold metastatic enrichment, CDKN1A co-expression reverses the EMT–cell-cycle anti-correlation, and drug-specific survival patterns reveal two distinct resistance strategies amenable to different therapeutic targeting. This framework is directly applicable to drug resistance studies in other cancer types where single-cell trajectory data are available.

## Methods

### Cell culture

PANC-1 cells (DSMZ, ACC-783) were maintained in Dulbecco’s Modified Eagle Medium (DMEM, high glucose, GlutaMAX; Gibco 61965059) supplemented with sodium pyruvate (Invitrogen P2256) and 10% fetal calf serum (FCS; PAN-Biotech P303306) in T-75 or T-150 flasks (TPP 90076/90151) at 37 °C and 5% CO₂, and passaged at 80–90% confluence. For passaging, cells were incubated for 3 min with 1% trypsin in PBS⁻ containing 0.5 mM EDTA (Sigma D8537, E5134), quenched with FCS-supplemented medium, centrifuged at 300 × g for 5 min, and resuspended to the desired volume. The genotypic and phenotypic characteristics of this line have been described previously^10^.

### Ethics and animal welfare

The 3D tumour tissue model is based on a decellularized porcine small intestinal submucosa (SISmuc) scaffold. All procedures involving porcine tissue were conducted in accordance with the German Animal Protection Law (§4 Abs. 3), FELASA, WHO and FDA guidelines, and were approved by the Ethics Committee of the District of Unterfranken (Regierung von Unterfranken), Würzburg, Germany, approval number 2532-2-12. Porcine jejunum was obtained as a by-product from animals slaughtered for food production; no animals were killed for the purpose of this study. No human participants or human material were involved in this study.

### Generation and culture of 3D SISmuc models

Decellularization of porcine jejunum followed the procedure of Linke^50^: intestinal segments were rinsed with PBS, decellularized with sodium deoxycholate monohydrate, rinsed again, and gamma-sterilized. SISmuc scaffolds were cut into ~15 × 15 mm squares and mounted between ring-like inserts (“cell crowns”) in 12-well plates (TPP 92012) in a transwell configuration, with 1.5 mL medium in the outer and 1.0 mL in the inner (luminal) compartment. PANC-1 cells were seeded on the luminal surface at 2 × 10⁵ cells/mL (500 µL); after 1 h of attachment, 1 mL of medium was added to each compartment. At each scheduled medium change, spent medium was removed with glass Pasteur pipettes and replaced with 1.0 mL (inner) and 1.5 mL (outer) fresh medium. The platform follows previously established protocols for tumour tissue engineering on SISmuc scaffolds^12,13^.

### Drug treatment and stimulation

Gemcitabine (GEM; Sigma G6423) was dissolved in sterile DMEM to 10 mM, diluted to 10 µM in complete medium, and applied to both compartments for 24 h on day 11 of culture. Recombinant human TGF-β1 (Peprotech 100-21) was reconstituted in PBS containing 1% BSA (Carl Roth 01633) to 10 µg/mL, diluted to 10 ng/mL immediately before use, and applied continuously from day 3 to day 14, with refreshing at each medium change. Four experimental conditions were generated: control, GEM, TGF-β1, and GEM + TGF-β1. An earlier iteration of this experimental system has been described previously^14^.

### Histology, immunofluorescence and viability assay

SISmuc constructs were fixed in 4% paraformaldehyde, paraffin-embedded, sectioned at 5 µm, deparaffinized and rehydrated. For haematoxylin and eosin staining, sections were stained with haematoxylin (Morphisto 61870036) for 6 min, blued under running water for 5 min, and counterstained with eosin (Morphisto 10177.01000) for 6 min, followed by dehydration through 70% and 96% ethanol, isopropanol (Carl Roth T910) and xylene (Carl Roth 9713), and mounting with Entellan (Merck 1079600500).

For Ki67 immunofluorescence, antigen retrieval was performed for 20 min at 100 °C in citrate buffer (pH 6.0) using a steam cooker (Braun FS20). A hydrophobic barrier was applied (ImmEdge pen, Vector Labs H-4000) and sections were blocked with 5% donkey serum (Sigma D9663) in antibody diluent (DCS AL120R500) for 20 min at room temperature. Primary anti-Ki67 antibody (Abcam ab1666) was applied at 1:100 overnight at 4 °C, followed by anti-rabbit Alexa Fluor 555 (Life Technologies A-31572) at 1:400 for 1 h at room temperature, protected from light. Sections were mounted with Fluoromount-G containing DAPI (Invitrogen SBA-0100-20). Ki67 is an established proliferation marker whose expression is tightly coupled to cell-cycle progression^51^.

For MTT viability assays, MTT solution (SERVA, 1 mg/mL) was added to the 3D models and incubated for 3 h. Medium was removed and formazan extracted with 2 mL isopropanol containing 0.04 N HCl on an orbital shaker for 1 h, repeated once for 30 min. Absorbance was measured at 570 nm (Tecan microplate reader, Männedorf).

### Single-cell suspension preparation and RNA sequencing

Constructs were washed three times with PBS⁻. Pre-warmed Accutase (1 mL; Sigma A6964) was applied to the luminal side for 30 min with trituration every 10 min, and the suspension was collected. Constructs were then treated with protease from *Bacillus licheniformis* (subtilisin A; Sigma P5380, 10 mg/mL) for 10 min with mixing at 5 min intervals, and crowns were rinsed twice with 1 mL PBS⁻. All fractions were pooled, adjusted to 5 mL and centrifuged at 300 × *g* for 5 min. Dead cells were removed (Miltenyi 130-090-101) and cell concentration adjusted according to 10x Genomics guidelines^52^.

Libraries were prepared at the HIRI Single-Cell Center (Würzburg) using 10x Genomics Single Cell 3′ v3 chemistry. Eight tagged samples were processed. FASTQ files were processed with Cell Ranger v9.0.0 against GRCh38 (RefSeq, October 2023) using the multi pipeline for demultiplexing and quantification, including intronic reads, and the aggregate command for merging per-sample outputs^52,53^. Technical parameters are reported in Supplementary Table 1.

### Preprocessing, quality control and clustering

Cell Ranger outputs were parsed with Pandas^54^ and SciPy^55^, assembled into per-sample AnnData objects and concatenated with Scanpy^56^ while preserving unique cell and gene identifiers. Gene symbols were mapped from Ensembl IDs via MyGene^57^, and CMO features were removed. Quality control thresholds were: mitochondrial RNA < 10%, ribosomal RNA < 50% (MT-, RPS- and RPL- prefixes), 1900–9000 detected genes, and 1–70,000 UMIs per cell. Doublets were removed with Scrublet^58^, and extreme outliers were excluded using a 5× median absolute deviation rule on log-transformed counts. Counts were normalized to 1 × 10⁴ UMIs per cell and log1p-transformed; the top 2000–4000 highly variable genes were selected using Scanpy default settings. Dimensionality reduction used PCA (ARPACK solver, 40 principal components) implemented with NumPy^59^ and SciPy^55^, followed by a k-nearest-neighbour graph (*k* = 8) and UMAP^60^. Leiden clustering^61^ was performed with a resolution sweep from 0 to 1 in steps of 0.01; one outlier cluster was removed and the graph and UMAP recomputed, yielding four stable clusters.

### Differential expression and pathway analysis

Differential expression was performed with the Scanpy rank_genes_groups function^56^ using a t-test, reporting log₂ fold change, raw *P* values and Benjamini–Hochberg FDR-adjusted *P* values. Gene set enrichment analysis used GSEApy^62^ on MSigDB Hallmark 2020 with 1000 permutations for cluster-level comparisons (minimum set size 10, maximum 50) and the log₂ ratio of classes as scoring metric. Pseudotime inference used scFates v1.0.6^63^ with a principal tree method on Palantir diffusion maps^64^ with n_eigs = 4; gene-trend clustering used n_neighbors = 50, correlation metric and resolution = 4. For trajectory-resolved enrichment, continuous pseudotime was binned at width 0.5 and intersected with Leiden clusters; bins containing fewer than 50 cells were merged. Per-bin GSEA used 100,000 permutations (minimum set size 10, maximum 100). Survival associations were assessed using Kaplan–Meier Plotter^65,66^.

### Dynamic network topology analysis

Directed, signed protein–protein interaction data were retrieved from OmniPath^67^ using the OmniPath Python client v1.0.8, filtered for directional and signed annotation, curation effort ≥3 and UniProt identifiers, and assembled in igraph v0.11.4^68^. The full reconstructed network comprised 2,588 nodes; for trajectory, knockout and shortest-path analyses the largest connected component (680 nodes, 1,403 edges) was used, preserving the edge type attribute (activation, inhibition or other). Expression-filtered subnetworks were constructed per pseudotime bin by retaining edges for which both endpoints were expressed (log read count >0.5), yielding 119–259 nodes and 128–362 edges per bin, with density 0.0053–0.0091 and average degree 2.15–2.80. Node and edge counts increased with pseudotime alongside slight density dilution, indicating progressively strengthened pathway connectivity. The interpretational limits of expression-derived network inference have been discussed elsewhere^69^.

Directed, shortest-path-based betweenness centrality was computed per trajectory subnetwork using igraph^68^ to track how regulatory bottlenecks shift across cell-state transitions, with temporal dynamics summarized by slope and Δ-change across ordered bins. For synergy quantification, the 20 most perturbed genes by |log₂FC| were designated the perturbed set in each pseudotime bin and perturbation condition. Three influence metrics were computed per node: the shortest-path betweenness fraction (the fraction of shortest paths between randomly sampled perturbed pairs passing through the node); Personalized PageRank (PPR) reach (steady-state probability summed over the perturbed set); and average shortest-path distance to perturbed nodes. A composite score was defined as PPR reach × |log₂FC | . Synergy was defined by Composite_Combined > Composite_TGFB + Composite_CC, PPR_Combined > PPR_TGFB + PPR_CC, and log₂FC > 0.

### CDK1 bottleneck and knockout analysis

CDK1 bottleneck regulators were identified by three complementary analyses. First, shortest-path overlap: on the latest S-phase plus TGF-β1 trajectory graph, all-pairs directed shortest-path distances were computed (OUT mode), and a pair (s,t) was considered CDK1-mediated if D(s,t) = D(s → CDK1) + D(CDK1→t); per-vertex overlap count and overlap fraction were reported. Second, a randomized knockout sweep: the knockout universe comprised all nodes in the trajectory graphs excluding CDK1 (UniProt P06493), which was never removed, reflecting its central role in cell-cycle control and cancer^29^. Neighbourhood expansion used OUT neighbours to radius r ∈ {0,1,2} on the global directed graph, with knockout set sizes m ∈ {1,2} and 4,000 trials per m using a fixed random seed. The per-trial effect was Δ = mean(B_CDK1^post) − mean(B_CDK1^baseline), with per-gene attribution Δ/m; the mean Δ across trials containing the seed and the fraction of trials in the top 1% by |Δ| were reported. Third, exact single-gene knockout: each seed was removed from every trajectory graph and CDK1 betweenness recomputed, giving a deterministic Δ betweenness. Regulators were ranked by a composite knockout score, a weighted z-sum of latest-bin overlap fraction (0.35), expected effect (0.35), top-1% hit rate (0.20) and single-knockout delta (0.10); higher scores indicate regulators that lie on CDK1-mediated paths and robustly reduce CDK1 betweenness when perturbed.

### PDAC atlas cross-examination

The PDAC single-cell atlas^15,70^ (726,107 cells, 231 patients, 12 studies) was converted from Seurat RDS to AnnData using SeuratDisk v0.0.0.9020 under R v4.3. The 300,577 ductal and cycling ductal cells were embedded with PHATE v1.0.11^71^ following PCA with 50 components, using *k* = 5, t = ‘auto’ and two components. PHATE preserves both local and global structure in single-cell data and is therefore well suited to visualising continuous progression arcs, in contrast to UMAP^60^, which prioritises local neighbourhood structure. Leiden signatures from the tissue model were scored per atlas cell as the mean log-normalized expression of the top marker genes per cluster (19 genes for L2); library-size correction was applied by linear regression of signature score on log(total UMI), with residuals used for all quantitative analyses.

CDK1, CDKN1A and WEE1 binary states were defined at >0 UMI in the raw count matrix, and eight combinatorial expression states were constructed. Metastatic enrichment was assessed by two-sided Fisher’s exact tests comparing metastatic with treatment-naïve primary tumours; pronounced microenvironmental heterogeneity between primary and metastatic PDAC lesions has been documented by spatial transcriptomic profiling^72^. EMT–cell-cycle coupling was quantified by Spearman correlation between aggregate EMT (FN1, VIM, SERPINE1, TGFBI, SNAI2, MMP2, THBS1) and cell-cycle (S-phase and G2/M gene sets) scores, stratified by CDK1/CDKN1A co-expression state (*n* > 4000 cells per group). Receiver operating characteristic analysis used scikit-learn v1.3.2 with 1000-iteration bootstrapped 95% confidence intervals across eight signatures. DUAL-program cells were defined as cells above the 75th percentile for both the L1 and L3 signature scores. All treatment-related comparisons are observational; batch effects were not re-corrected beyond the original atlas integration. No multiple-testing correction was applied to the primary axis-state comparisons, which were hypothesis-driven. All statistical tests were two-sided.

### Software and reproducibility

Analyses used Python v3.10 with scanpy v1.9.6^56^, scFates v1.0.6^63^, Palantir v1.3.3^64^, phate v1.0.11^71^, scikit-learn v1.3.2, scipy v1.11^55^, numpy v1.24^59^, igraph v0.11.4^68^, NetworkX, GSEApy^62^, matplotlib v3.8^73^, seaborn^74^ and pandas^54^, and R v4.3 with SeuratDisk v0.0.0.9020. Random seeds were set throughout for reproducibility (seed = 42 for principal tree inference; fixed random number generator for knockout sweeps). All Jupyter notebooks, conda environment YAML files, Cell Ranger configuration CSVs, intermediate H5AD files and figure-generation code are available as described in the Code Availability statement.

## Supplementary information

Supplementary Information

Supplementary table S1

## Data Availability

The raw single-cell RNA sequencing data generated in this study have been deposited in ArrayExpress under accession number E-MTAB-17360 (https://www.ebi.ac.uk/biostudies/arrayexpress/studies/E-MTAB-17360). The processed annotated dataset will be made available via the EMBL-EBI Single Cell Expression Atlas. The PDAC single-cell atlas used for external contextualization is publicly available from Zenodo (https://doi.org/10.5281/zenodo.14199536).

## Code availability

Analysis code and conda environment YAML files required to reproduce all results are available at https://github.com/johannes-balkenhol/PANC_3D-tissue_scAnalysis and archived on Zenodo under https://doi.org/10.5281/zenodo.21353935 (concept DOI, resolving to the latest version; version 3D-tissue-model: https://doi.org/10.5281/zenodo.21353936), released under the MIT License. Additional preprocessing code is available at https://github.com/Almasimaryam/scRNAseq-pipeline-Py.

## References

[1] Siegel, R. L., Giaquinto, A. N. & Jemal, A. Cancer statistics, 2024. *CA Cancer J. Clin.* **74**, 12–49 (2024). 10.3322/caac.21820 38230766

[2] Conroy, T. et al. FOLFIRINOX versus Gemcitabine for metastatic pancreatic cancer. *N. Engl. J. Med.* **364**, 1817–1825 (2011). 10.1056/NEJMoa1011923 21561347

[3] Sohal, D. P. S. et al. Metastatic pancreatic cancer: ASCO clinical practice guideline update. *J. Clin. Oncol.* **36**, 2545–2556 (2018). 10.1200/JCO.2018.78.9636 PMC7504972 29791286

[4] Hosein, A. N. et al. Translational advances in pancreatic ductal adenocarcinoma therapy. *Nat. Cancer* **3**, 272–286 (2022). 10.1038/s43018-022-00349-2 35352061

[5] Conroy, T. et al. FOLFIRINOX or Gemcitabine as adjuvant therapy for pancreatic cancer. *N. Engl. J. Med.* **379**, 2395–2406 (2018). 10.1056/NEJMoa1809775 30575490

[6] Conroy, T. et al. Five-year outcomes of FOLFIRINOX vs Gemcitabine as adjuvant therapy for pancreatic cancer: a randomized clinical trial. *JAMA Oncol.* **8**, 1571–1578 (2022). 10.1001/jamaoncol.2022.3829 PMC9437831 36048453

[7] Cappella, P. et al. Cell cycle effects of Gemcitabine. *Int. J. Cancer* **93**, 401–408 (2001). 10.1002/ijc.1351 11433406

[8] de Sousa Cavalcante, L. & Monteiro, G. Gemcitabine: metabolism and molecular mechanisms of action, sensitivity and chemoresistance in pancreatic cancer. *Eur. J. Pharmacol.* **741**, 8–16 (2014). 10.1016/j.ejphar.2014.07.041 25084222

[9] Neesse, A. et al. Stromal biology and therapy in pancreatic cancer. *Gut* **60**, 861–868 (2011). 10.1136/gut.2010.226092 20966025

[10] Deer, E. L. et al. Phenotype and genotype of pancreatic cancer cell lines. *Pancreas* **39**, 425–435 (2010). 10.1097/MPA.0b013e3181c15963 PMC2860631 20418756

[11] Striefler, J. K. et al. P53 overexpression and Ki67-index are associated with outcome in ductal pancreatic adenocarcinoma with adjuvant gemcitabine treatment. *Pathol. Res. Pract.* **212**, 726–734 (2016). 10.1016/j.prp.2016.06.001 27461834

[12] Göttlich, C. et al. A combined tissue-engineered/in silico signature tool patient stratification in lung cancer. *Mol. Oncol.* **12**, 1264–1285 (2018). 10.1002/1878-0261.12323 PMC6068345 29797762

[13] Peindl, M. et al. EMT, stemness, and drug resistance in biological context: a 3D tumor tissue/in silico platform for analysis of combinatorial treatment in NSCLC with aggressive KRAS-biomarker signatures. *Cancers* **14**, 2176 (2022). 10.3390/cancers14092176 PMC9099837 35565305

[14] Caliskan, A. et al. Mathematical strategies for predicting resistant subpopulations from scRNAseq data of a PANC-1 3D tissue model: Insight into gemcitabine resistance and TGFB1-induced invasion and EMT. *Comput. Struct. Biotechnol. J.* **27**, 4476–4495 (2025). 10.1016/j.csbj.2025.10.032 PMC12593213 41209344

[15] Loveless, I. M. et al. Human pancreatic cancer single-cell atlas reveals association of CXCL10+ fibroblasts and basal subtype tumor cells. *Clin. Cancer Res.* **31**, 756–772 (2025). 10.1158/1078-0432.CCR-24-2183 PMC11831110 39636224

[16] Namima, D. et al. The effect of Gemcitabine on cell cycle arrest and microRNA signatures in pancreatic cancer cells. *In Vivo* **34**, 3195–3203 (2020). 10.21873/invivo.12155 PMC7811671 33144424

[17] Shewach, D. S. & Lawrence, T. S. Gemcitabine and radiosensitization in human tumor cells. *Invest. N. Drugs* **14**, 257–263 (1996). 10.1007/BF00194528 8958180

[18] Wang, Y. et al. Centrosome-associated regulators of the G2/M checkpoint as targets for cancer therapy. *Mol. Cancer* **8**, 8 (2009). 10.1186/1476-4598-8-8 PMC2657106 19216791

[19] Marumoto, T., Zhang, D. & Saya, H. Aurora-A—a guardian of poles. *Nat. Rev. Cancer* **5**, 42–50 (2005). 10.1038/nrc1526 15630414

[20] Lens, S. M. A., Voest, E. E. & Medema, R. H. Shared and separate functions of polo-like kinases and aurora kinases in cancer. *Nat. Rev. Cancer* **10**, 825–841 (2010). 10.1038/nrc2964 21102634

[21] Guenther, M. et al. TPX2 expression as a negative predictor of gemcitabine efficacy in pancreatic cancer. *Br. J. Cancer* **129**, 175–182 (2023). 10.1038/s41416-023-02295-x PMC10307892 37142730

[22] Davidson, J. D. et al. An increase in the expression of ribonucleotide reductase large subunit 1 is associated with gemcitabine resistance in non-small cell lung cancer cell lines. *Cancer Res.* **64**, 3761–3766 (2004). 10.1158/0008-5472.CAN-03-3363 15172981

[23] Jordheim, L. P. & Dumontet, C. Review of recent studies on resistance to cytotoxic deoxynucleoside analogues. *Biochim. Biophys. Acta* **1776**, 138–159 (2007). 10.1016/j.bbcan.2007.07.004 17881132

[24] Zhan, Y. et al. Inhibiting RRM2 to enhance the anticancer activity of chemotherapy. *Biomed. Pharmacother.* **133**, 110996 (2021). 10.1016/j.biopha.2020.110996 33227712

[25] Li, F. & Ling, X. Survivin study: an update of “what is the next wave”? *J. Cell. Physiol.* **208**, 476–486 (2006). 10.1002/jcp.20634 PMC2821201 16557517

[26] Kagawa, S. et al. Akt/mTOR signaling pathway is crucial for gemcitabine resistance induced by Annexin II in pancreatic cancer cells. *J. Surg. Res.* **178**, 758–767 (2012). 10.1016/j.jss.2012.05.065 22726648

[27] Son, B. et al. Targeted therapy of cancer stem cells: inhibition of mTOR in preclinical and clinical research. *Cell Death Dis.* **15**, 696 (2024). 10.1038/s41419-024-07077-8 PMC11442590 39349424

[28] Chawsheen, M. A. & Dash, P. R. mTOR modulates resistance to gemcitabine in lung cancer in an MTORC2 dependent mechanism. *Cell. Signal.* **81**, 109934 (2021). 10.1016/j.cellsig.2021.109934 33545231

[29] Wang, Q., Bode, A. M. & Zhang, T. Targeting CDK1 in cancer: mechanisms and implications. *npj Precis. Oncol.* **7**, 58 (2023). 10.1038/s41698-023-00407-7 PMC10264400 37311884

[30] Chen, P. et al. Gemcitabine resistance mediated by ribonucleotide reductase M2 in lung squamous cell carcinoma is reversed by GW8510 through autophagy induction. *Clin. Sci.* **132**, 1417–1433 (2018). 10.1042/CS20180010 29853661

[31] Chen, G. et al. PLAU promotes cell proliferation and epithelial–mesenchymal transition in head and neck squamous cell carcinoma. *Front. Genet.* **12**, 651882 (2021). 10.3389/fgene.2021.651882 PMC8173099 34093649

[32] Mahon, P. C. et al. S100A4 contributes to the suppression of BNIP3 expression, chemoresistance, and inhibition of apoptosis in pancreatic cancer. *Cancer Res.* **67**, 6786–6795 (2007). 10.1158/0008-5472.CAN-07-0440 17638890

[33] Xavier, C. P. R. et al. Chitinase 3-like-1 and fibronectin in the cargo of extracellular vesicles shed by human macrophages influence pancreatic cancer cellular response to Gemcitabine. *Cancer Lett.* **501**, 210–223 (2021). 10.1016/j.canlet.2020.11.013 33212158

[34] Du, Y. et al. E3 ubiquitin ligase UBR5 promotes gemcitabine resistance in pancreatic cancer by inducing O-GlcNAcylation-mediated EMT via destabilization of OGA. *Cell Death Dis.* **15**, 340 (2024). 10.1038/s41419-024-06729-z PMC11099055 38755129

[35] Li, Z. et al. Identification of MBOAT2 as an unfavorable biomarker correlated with KRAS activation and reduced CD8+ T-cell infiltration in pancreatic cancer. *J. Oncol.* **2022**, 4269733 (2022). 10.1155/2022/4269733 PMC9095372 35571489

[36] Lovisa, S. et al. Epithelial-to-mesenchymal transition induces cell cycle arrest and parenchymal damage in renal fibrosis. *Nat. Med.* **21**, 998–1009 (2015). 10.1038/nm.3902 PMC4587560 26236991

[37] Akhmetkaliyev, A., Alibrahim, N., Shafiee, D. & Tulchinsky, E. EMT/MET plasticity in cancer and Go-or-Grow decisions in quiescence: the two sides of the same coin? *Mol. Cancer* **22**, 90 (2023). 10.1186/s12943-023-01793-z PMC10230810 37259089

[38] Melisi, D. et al. Galunisertib plus gemcitabine vs. gemcitabine for first-line treatment of patients with unresectable pancreatic cancer. *Br. J. Cancer* **119**, 1208–1214 (2018). 10.1038/s41416-018-0246-z PMC6251034 30318515

[39] Musa, J., Aynaud, M. M., Mirabeau, O., Delattre, O. & Grünewald, T. G. MYBL2 (B-Myb): a central regulator of cell proliferation, cell survival and differentiation involved in tumorigenesis. *Cell Death Dis.* **8**, e2895 10.1038/cddis.2017.244 (2017). PMC5520903 28640249

[40] Datto, M. B. et al. Transforming growth factor beta induces the cyclin-dependent kinase inhibitor p21 through a p53-independent mechanism. *Proc. Natl. Acad. Sci. USA* **92**, 5545–5549 (1995). 10.1073/pnas.92.12.5545 PMC41732 7777546

[41] Debaugnies, M. et al. RHOJ controls EMT-associated resistance to chemotherapy. *Nature* **616**, 168–175 (2023). 10.1038/s41586-023-05838-7 PMC10076223 36949199

[42] Schuhwerk, H. & Brabletz, T. Mutual regulation of TGFβ-induced oncogenic EMT, cell cycle progression and the DDR. *Semin. Cancer Biol.* **97**, 86–103 (2023). 10.1016/j.semcancer.2023.11.009 38029866

[43] Werba, G. et al. Single-cell RNA sequencing reveals the effects of chemotherapy on human pancreatic adenocarcinoma and its tumor microenvironment. *Nat. Commun.* **14**, 797 (2023). 10.1038/s41467-023-36296-4 PMC9925748 36781852

[44] Parsels, L. A. et al. Dissociation of gemcitabine chemosensitization by CHK1 inhibition from cell cycle checkpoint abrogation and aberrant mitotic entry. *Cell Cycle* **15**, 730–739 (2016). 10.1080/15384101.2016.1148841 PMC4845922 26890478

[45] Karnitz, L. M. & Zou, L. Molecular pathways: targeting ATR in cancer therapy. *Clin. Cancer Res.* **21**, 4780–4785 (2015). 10.1158/1078-0432.CCR-15-0479 PMC4631635 26362996

[46] Cuneo, K. C. et al. Dose escalation trial of the Wee1 inhibitor adavosertib (AZD1775) in combination with gemcitabine and radiation for patients with locally advanced pancreatic cancer. *J. Clin. Oncol.* **37**, 2643–2650 (2019). 10.1200/JCO.19.00730 PMC7006846 31398082

[47] Jones, R. et al. A phase I/II trial of oral SRA737 (a Chk1 inhibitor) given in combination with low-dose gemcitabine in patients with advanced cancer. *Clin. Cancer Res.* **29**, 331–340 (2023). 10.1158/1078-0432.CCR-22-2074 PMC10539020 36378548

[48] Salvador-Barbero, B. et al. CDK4/6 inhibitors impair recovery from cytotoxic chemotherapy in pancreatic adenocarcinoma. *Cancer Cell* **37**, 340–353 (2020). 10.1016/j.ccell.2020.01.007 32109375

[49] Bromberg, J. F. et al. Stat3 as an oncogene. *Cell* **98**, 295–303 (1999). 10.1016/s0092-8674(00)81959-5 10458605

[50] Linke, K. et al. Engineered liver-like tissue on a capillarized matrix for applied research. *Tissue Eng.* **13**, 2699–2707 (2007). 10.1089/ten.2006.0388 17867928

[51] Uxa, S. et al. Ki-67 gene expression. *Cell Death Differ.* **28**, 3357–3370 (2021). 10.1038/s41418-021-00823-x PMC8629999 34183782

[52] 10x Genomics. Cell Ranger User Guide. https://www.10xgenomics.com/support/software/cell-ranger/latest (2024).

[53] 10x Genomics. Single-cell gene expression software pipelines: choosing a pipeline. https://www.10xgenomics.com/support/software/cell-ranger/latest/analysis/running-pipelines/cr-choosing-a-pipeline (2024).

[54] McKinney, W. Data structures for statistical computing in Python. *Proc. 9th Python Sci. Conf.* **51**, 56 (2010).

[55] Virtanen, P. et al. SciPy 1.0: fundamental algorithms for scientific computing in Python. *Nat. Methods* **17**, 261–272 (2020). 10.1038/s41592-019-0686-2 PMC7056644 32015543

[56] Wolf, F. A., Angerer, P. & Theis, F. J. SCANPY: large-scale single-cell gene expression data analysis. *Genome Biol.* **19**, 15 (2018). 10.1186/s13059-017-1382-0 PMC5802054 29409532

[57] Xin, J. et al. High-performance web services for querying gene and variant annotation. *Genome Biol.* **17**, 91 (2016). 10.1186/s13059-016-0953-9 PMC4858870 27154141

[58] Wolock, S. L., Lopez, R. & Klein, A. M. Scrublet: computational identification of cell doublets in single-cell transcriptomic data. *Cell Syst.* **8**, 281–291 (2019). 10.1016/j.cels.2018.11.005 PMC6625319 30954476

[59] Harris, C. R. et al. Array programming with NumPy. *Nature* **585**, 357–362 (2020). 10.1038/s41586-020-2649-2 PMC7759461 32939066

[60] McInnes, L., Healy, J. & Melville, J. UMAP: uniform manifold approximation and projection for dimension reduction. Preprint at https://arxiv.org/abs/1802.03426 (2018).

[61] Traag, V. A., Waltman, L. & van Eck, N. J. From Louvain to Leiden: guaranteeing well-connected communities. *Sci. Rep.* **9**, 5233 (2019). 10.1038/s41598-019-41695-z PMC6435756 30914743

[62] Fang, Z., Liu, X. & Peltz, G. GSEApy: a comprehensive package for performing gene set enrichment analysis in Python. *Bioinformatics* **39**, btac757 10.1093/bioinformatics/btac757 (2023). PMC9805564 36426870

[63] Faure, L., Soldatov, R., Kharchenko, P. V. & Adameyko, I. scFates: a scalable Python package for advanced pseudotime and bifurcation analysis from single-cell data. *Bioinformatics* **39**, btac746 10.1093/bioinformatics/btac746 (2023). PMC9805561 36394263

[64] Setty, M. et al. Characterization of cell fate probabilities in single-cell data with Palantir. *Nat. Biotechnol.* **37**, 451–460 (2019). 10.1038/s41587-019-0068-4 PMC7549125 30899105

[65] Gyorffy, B. Integrated analysis of public datasets for the discovery and validation of survival-associated genes in solid tumors. *Innovation* **5**, 100625 (2024). 10.1016/j.xinn.2024.100625 PMC11066458 38706955

[66] Gyorffy, B. Transcriptome-level discovery of survival-associated biomarkers and therapy targets in non-small-cell lung cancer. *Br. J. Pharmacol.* **181**, 362–374 (2024). 10.1111/bph.16257 37783508

[67] Türei, D., Korcsmáros, T. & Saez-Rodriguez, J. OmniPath: guidelines and gateway for literature-curated signaling pathway resources. *Nat. Methods* **13**, 966–967 (2016). 10.1038/nmeth.4077 27898060

[68] Csardi, G. & Nepusz, T. The igraph software package for complex network research. InterJournal Complex Syst. 1695 (2006).

[69] Serin, E. A. R., Nijveen, H., Hilhorst, H. W. M. & Ligterink, W. Learning from co-expression networks: possibilities and challenges. *Front. Plant Sci.* **7**, 444 (2016). 10.3389/fpls.2016.00444 PMC4825623 27092161

[70] Loveless, I. M. & Steele, N. G. Single Cell RNAseq Pancreatic Cancer Atlas. Zenodo 10.5281/zenodo.14199536 (2024).

[71] Moon, K. R. et al. Visualizing structure and transitions in high-dimensional biological data. *Nat. Biotechnol.* **37**, 1482–1492 (2019). 10.1038/s41587-019-0336-3 PMC7073148 31796933

[72] Khaliq, A. M. et al. Spatial transcriptomic analysis of primary and metastatic pancreatic cancers highlights tumor microenvironmental heterogeneity. *Nat. Genet.* **56**, 2455–2465 (2024). 10.1038/s41588-024-01914-4 39294496

[73] Hunter, J. D. Matplotlib: a 2D graphics environment. *Comput. Sci. Eng.* **9**, 90–95 (2007).

[74] Waskom, M. seaborn: statistical data visualization. *J. Open Source Softw.* **6**, 3021 (2021).

