# Supplementary material for: 3D tissue model to patient atlas: dynamic network topology reveals PDAC chemoresistance bottlenecks
Source: NPJ Syst Biol Appl. 2026 Oct 23;12:158. doi: 10.1038/s41540-026-00830-z (PMC13694714; doi:10.1038/s41540-026-00830-z)
Supplement: Supplementary file 1 [file 41540_2026_830_MOESM1_ESM.pdf]

Supplement for "3D tissue model to patient atlas: dynamic network topology reveals PDAC chemoresistance bottlenecks"

Maryam Almasi[1,*], Jesús Guillermo Nieves Pereira[2,*], Thomas Dandekar[1,**], Gudrun Dandekar[2,**], Johannes Balkenhol[1,**]

[1]Department of Bioinformatics, Biocenter, University of Würzburg, Am Hubland, 97074 Würzburg, Germany, [2]Department for Functional Materials in Medicine and Dentistry, University Hospital Würzburg, Röntgenring 11, 97070 Würzburg, Germany

[*]These authors contributed equally to this work as first authors.
[**]These authors contributed equally to this work as senior authors.

Contents

Supplementary Figures....3

Supplementary Figure 1. Ribosome counts and UMAP clustering of single-cell pancreatic cancer data....3

Supplementary Figure 2. Gene set enrichment and differential expression analysis across leiden clusters....4

Supplementary Figure 3. Gene set enrichment and differential expression analysis of GEM-surviving cells....5

Supplementary Figure 4. Cell cycle–associated gene expression and phase distribution across clusters and treatments....6

Supplementary Figure 5. Trajectory inference indicates progression of cell states under different treatment conditions....7

Supplementary Figure 6. Temporal transition profiles in 3D PANC tissues. 8

Supplementary Figure 7. Key marker genes defining distinct cell states associated with poor clinical prognosis....8

Supplementary Figure 8. CDK1 downstream targets associated with gemcitabine resistance and poor prognosis....10

Supplementary Figure 9. Betweenness centrality across the dynamic regulatory network identifies CDK1 as a central bottleneck....11

Supplementary Figure 10. Molecular gate schematic of the CDK1–CDKN1A–WEE1 bottleneck....12

Supplementary Figure 11. Multi-level mapping of CDK1–CDKN1A–WEE1 bottleneck signatures in the PDAC single-cell atlas....13

Supplementary Note 1. Evaluation of systemic single-cell analysis approaches21....

Supplementary Note 2. Tissue surface and core23....................................
Supplementary References25...

Supplementary Figures

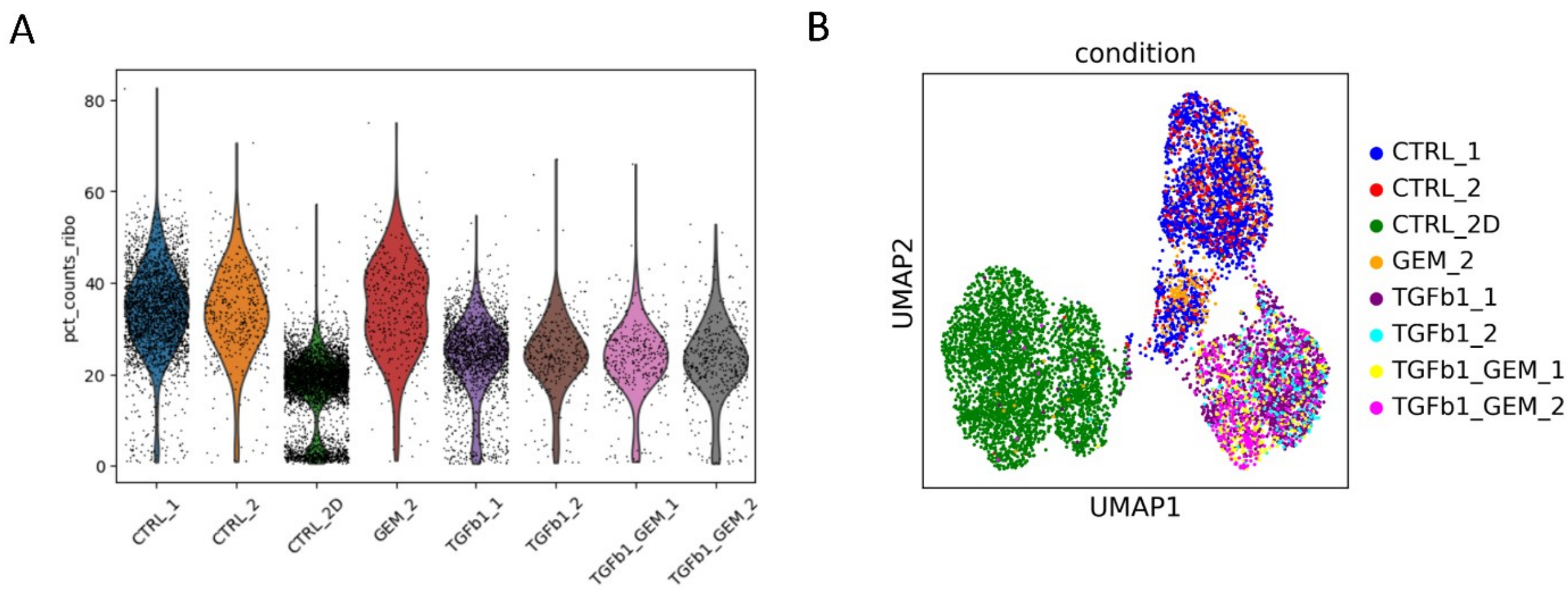

Supplementary Figure 1. Ribosome counts and UMAP clustering of single-cell pancreatic cancer data. (A) Distribution of ribosome content across control, GEM-treated, and TGFβ1-induced cells. (B) UMAP projections indicate distinct clustering: 2D and 3D control tissues form separate clusters from TGFβ1-induced tissues. Notably, GEM-treated cells do not form new clusters but rather integrate into the existing control or control + TGFβ1 clusters; furthermore, GEM-treated cells are absent in 2D tissues.

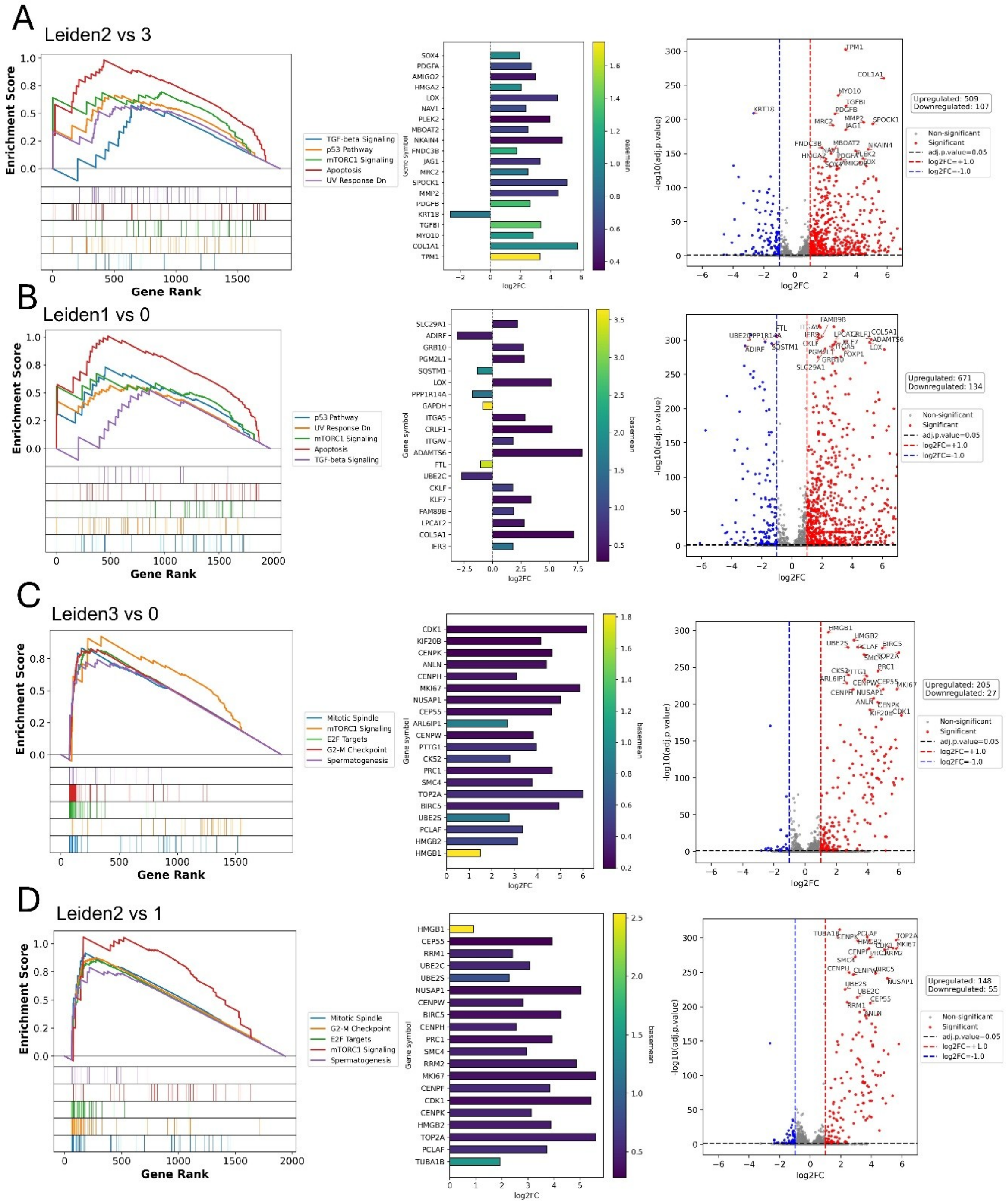

Supplementary Figure 2. Gene set enrichment and differential expression analysis across leiden clusters. (A) Pathway enrichment analysis summarizing key signaling differences between Leiden clusters. (B) Differential gene expression heatmaps across Leiden cluster comparisons. (C) Volcano plots depicting the differential expression profiles among clusters, highlighting substantial heterogeneity in pathway activation and gene expression.

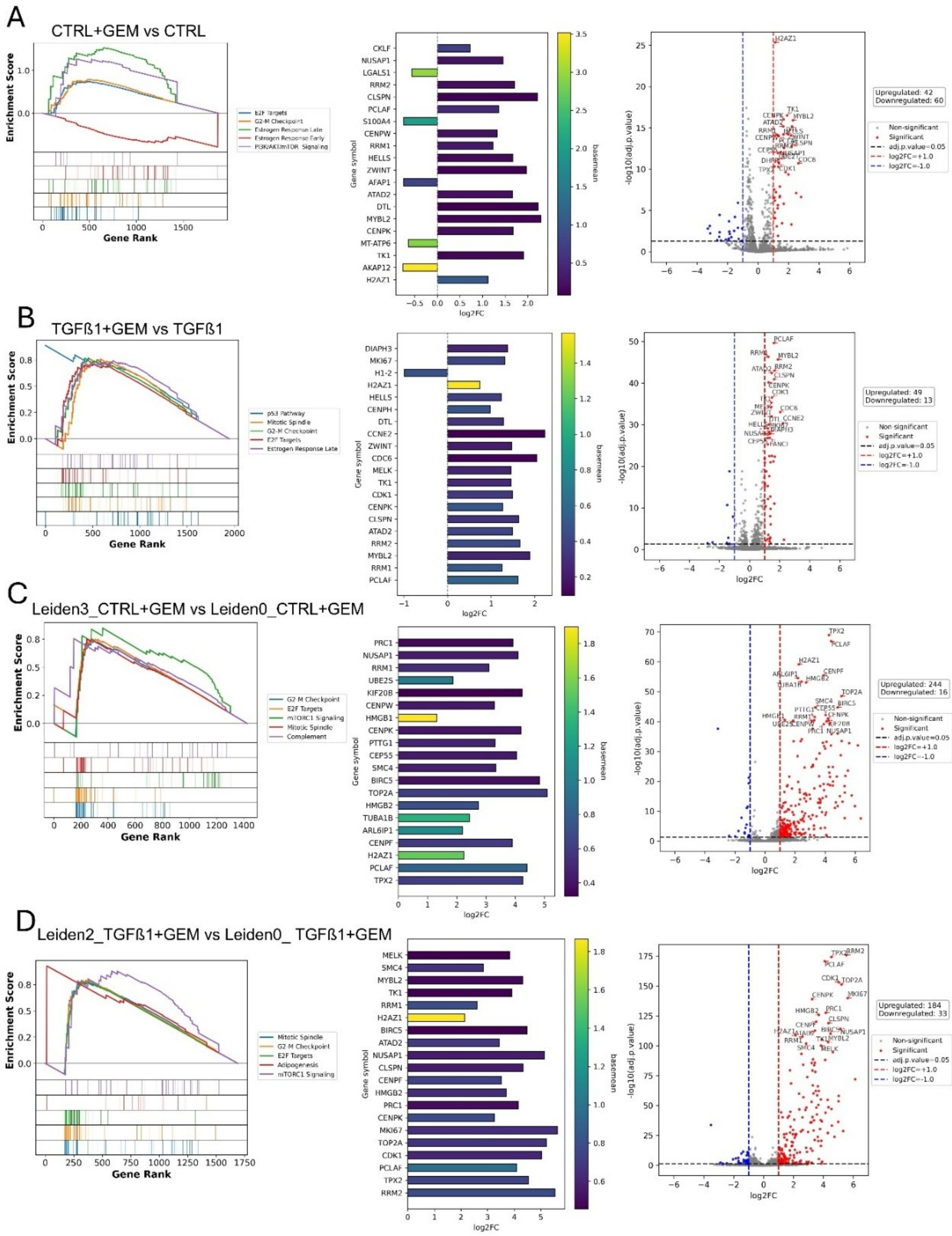

Supplementary Figure 3. Gene set enrichment and differential expression analysis of GEM-surviving cells. (A) Pathway enrichment analysis comparing GEM-surviving cells across treatment conditions. (B) Differential gene expression heatmaps for GEM-surviving cell populations. (C) Volcano plots displaying the differential expression profiles, highlighting substantial heterogeneity in pathway activation and gene expression among GEM-surviving cells.

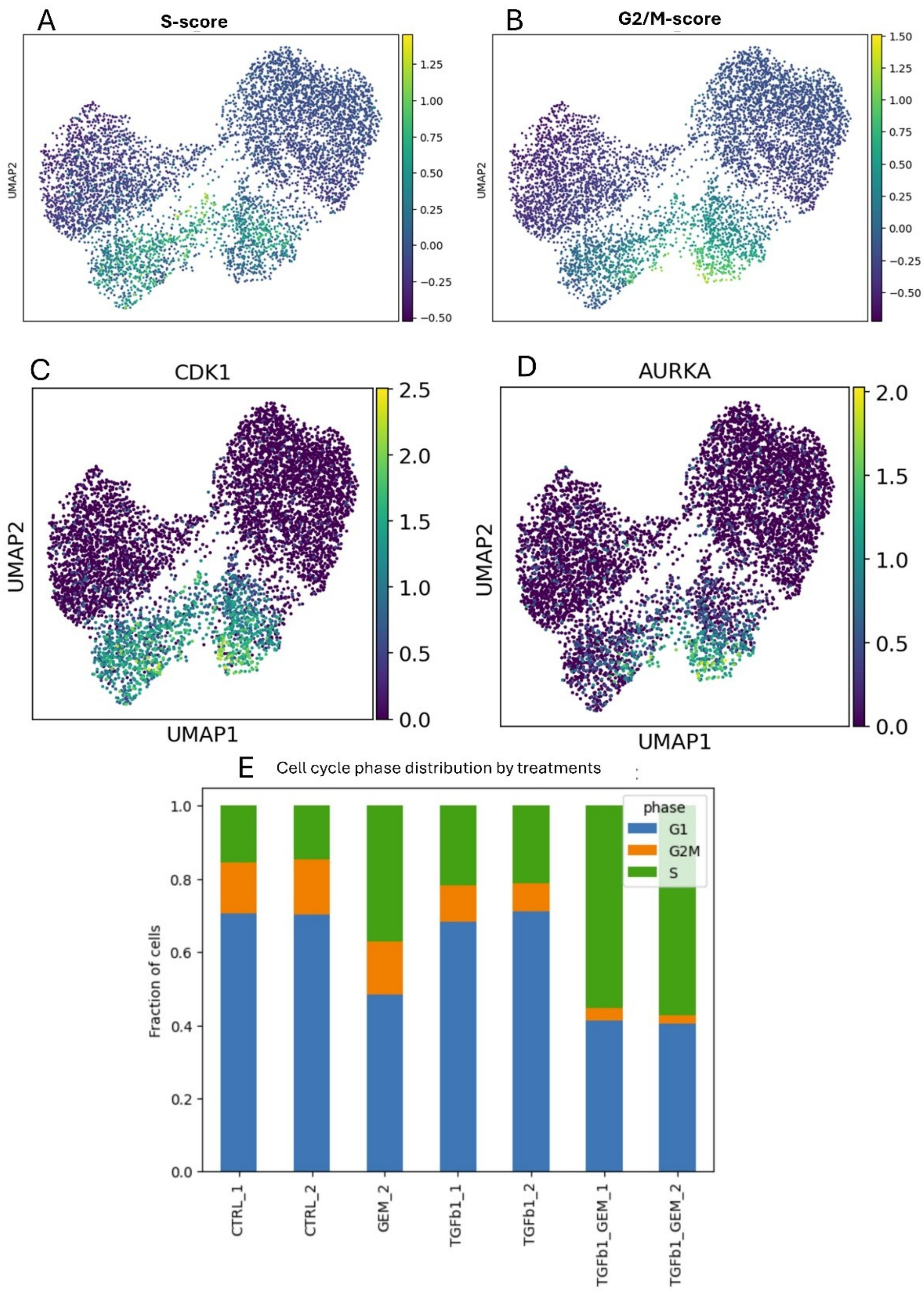

Supplementary Figure 4. Cell cycle–associated gene expression and phase distribution across clusters and treatments. (A–D) UMAP plots showing expression of key cell cycle–related genes: (A) S-score (S-phase signature), (B) G2M-score (G2/M signature), (C) CDK1, and (D) AURKA. Cells with high expression of these genes are enriched in specific regions of the embedding corresponding to proliferative states. (E) Cell cycle phase composition by treatment group, indicating differences in proliferative states between control, TGFβ1, GEM, and TGFβ1 + GEM conditions.

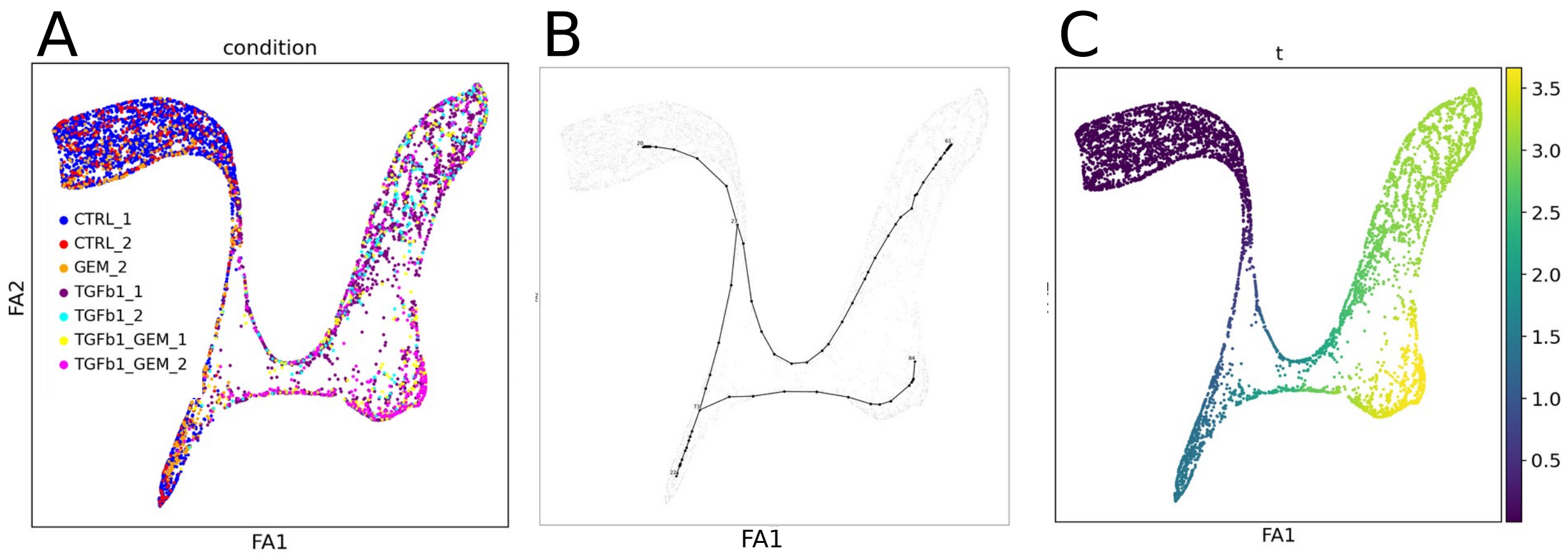

Supplementary Figure 5. Trajectory inference indicates progression of cell states under different treatment conditions. (A) Force-directed layout (FA) plot colored by experimental condition, showing the distribution of CTRL, TGFβ1, GEM, and TGFβ1 + GEM cells along the inferred trajectory. (B) Monocle3-derived pseudotime trajectory with root and branch points, indicating the inferred lineage relationships between cell states. (C) Pseudotime values mapped onto the FA plot, illustrating the gradual progression from early to late cell states along the trajectory.

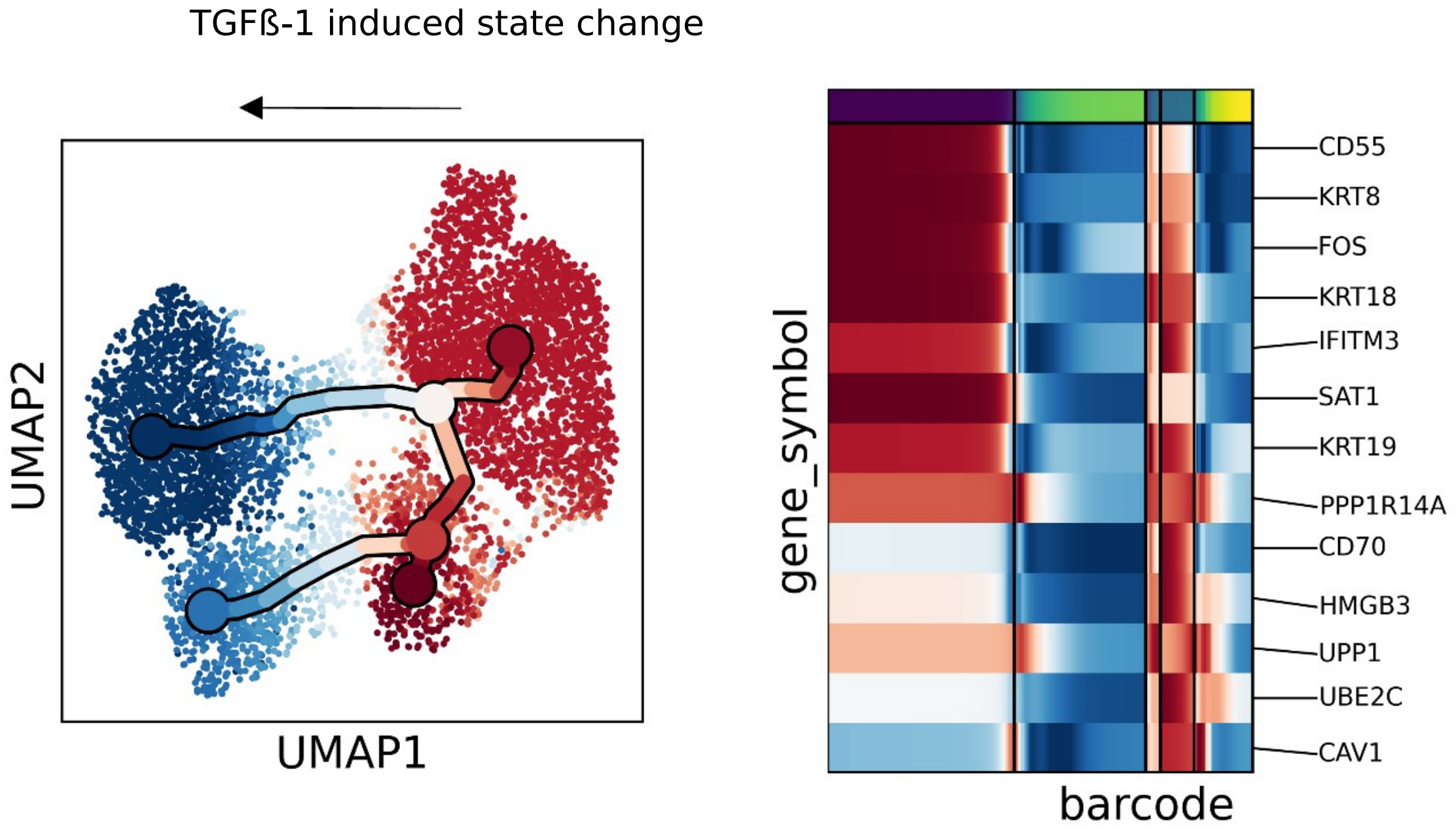

Supplementary Figure 6. Temporal transition profiles in 3D PANC tissues. Cluster analysis identifies a subset of cells with reduced expression of epithelial markers, including KRT18 and KRT19, in response to TGFβ1 induction.

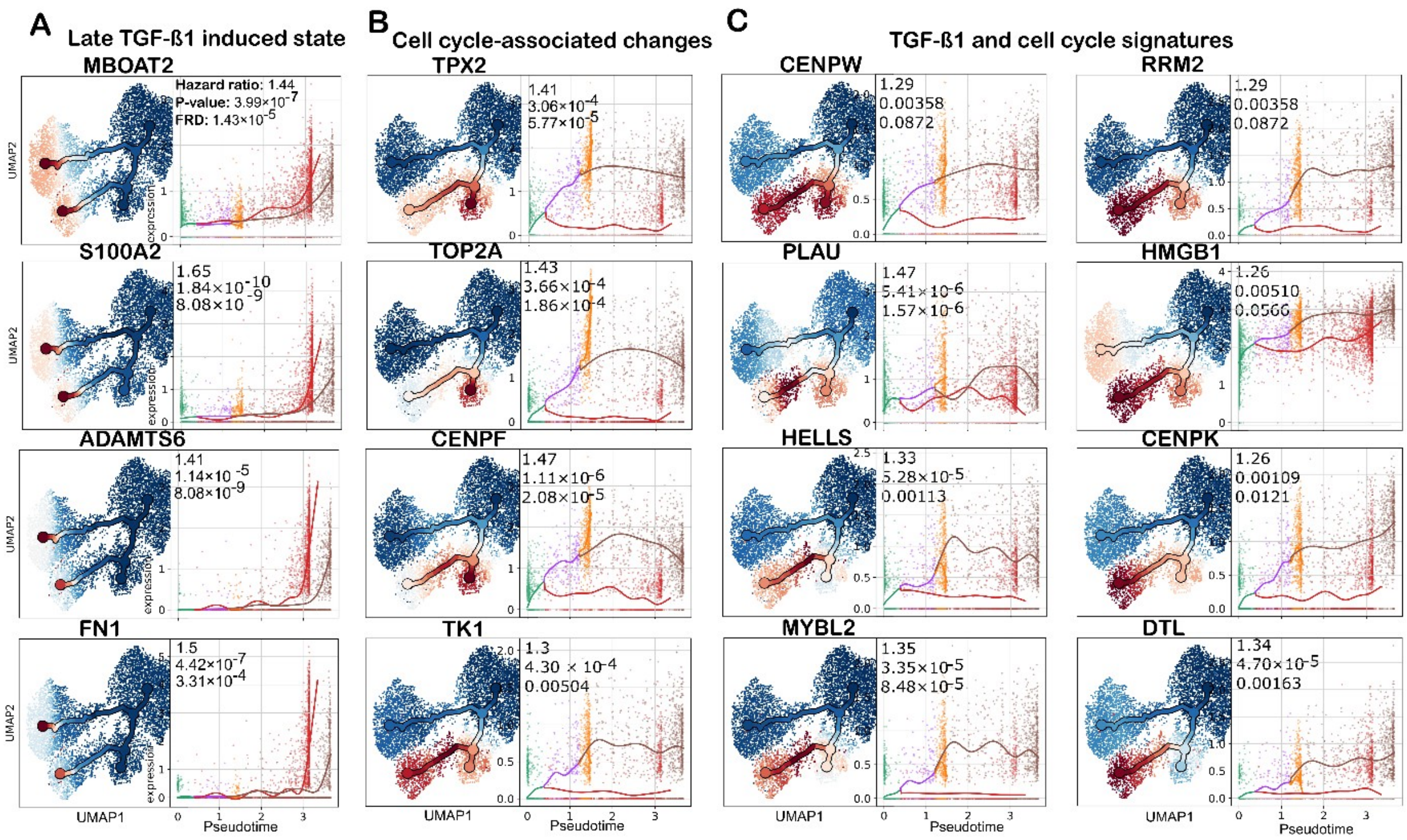

Supplementary Figure 7. Key marker genes defining distinct cell states associated with poor clinical prognosis.
(A–C) UMAP and pseudo-time plots showing the expression patterns of selected genes representing

distinct transcriptional modules in 3D-cultured PANC-1 cells following TGF-β1 stimulation. Each gene panel includes a UMAP embedding (left) with cells colored by fitted expression values (red = high, blue = low) and the corresponding pseudo-time trend plot (right) showing gene expression dynamics along the inferred trajectory branches. Each color in the pseudo-time plots corresponds to a milestone along the Leiden-defined clusters: green – early milestone (Leiden 0), orange – intermediate milestone (Leiden 3), red – late milestone (Leiden 1), and brown – late milestone (Leiden 2). Solid colored lines represent the average smoothed expression trends for each branch. (A) Representative marker genes exhibiting late-onset activation following TGF-β1–induced state transitions. (B) Genes associated with cell cycle–driven changes, marking G1 → S phase progression. (C) Genes showing combined expression signatures of TGF-β1 induction and cell cycle regulation, reflecting overlapping transcriptional programs that sustain these transition processes. Hazard ratios (HR), P-values, and FDR-adjusted significance levels from Kaplan–Meier survival analyses are provided to indicate associations with clinical outcomes.

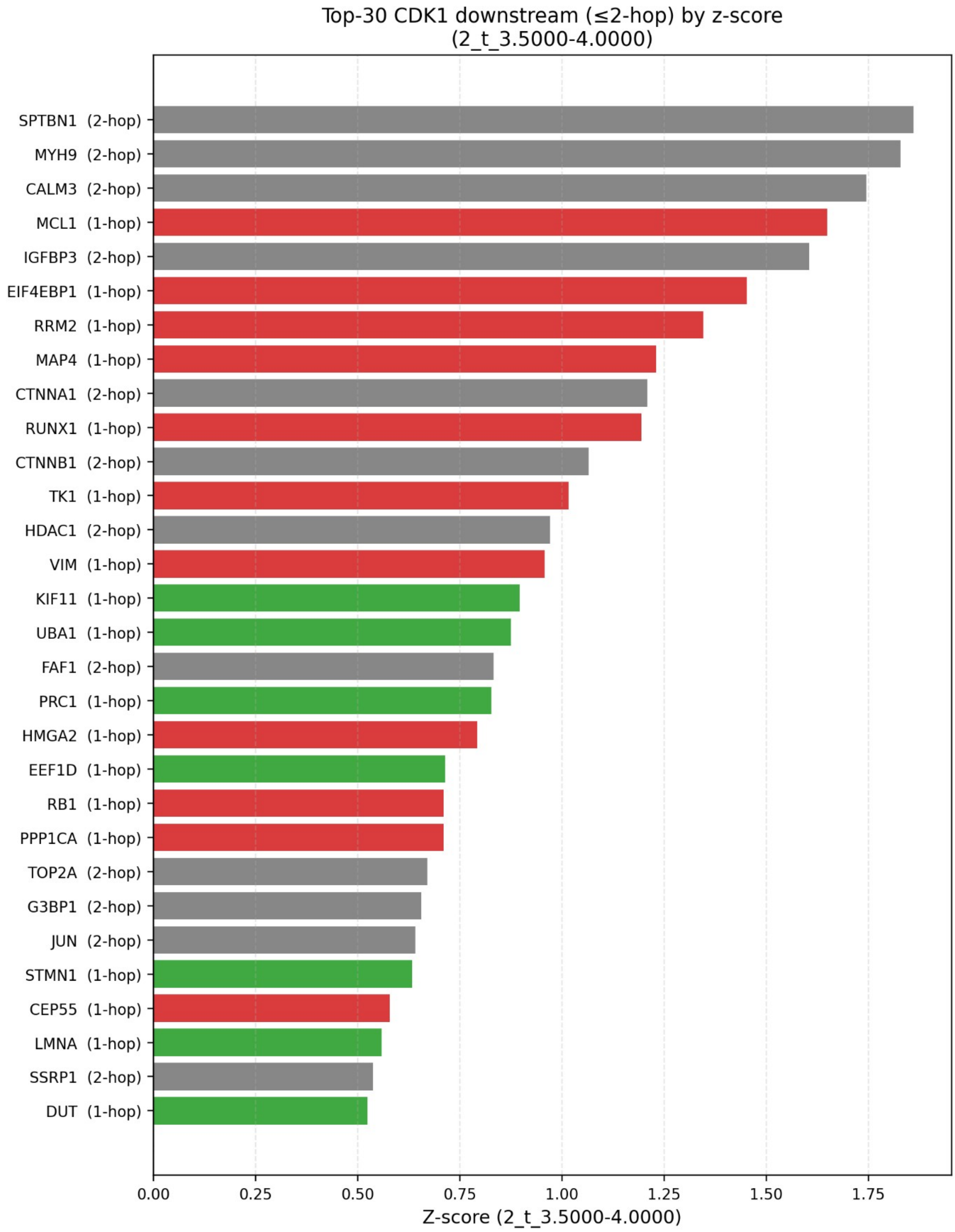

Supplementary Figure 8. CDK1 downstream targets associated with gemcitabine resistance and poor prognosis.

Network visualization of CDK1 and its downstream interaction partners identified through centrality analysis. Nodes represent genes with significant expression changes in the combined S-phase + TGFβ1 state; edges indicate known protein–protein interactions. Highlighted targets include key regulators of cell-cycle progression, DNA damage response, and replication stress tolerance linked to chemoresistance and adverse clinical outcomes in pancreatic cancer.

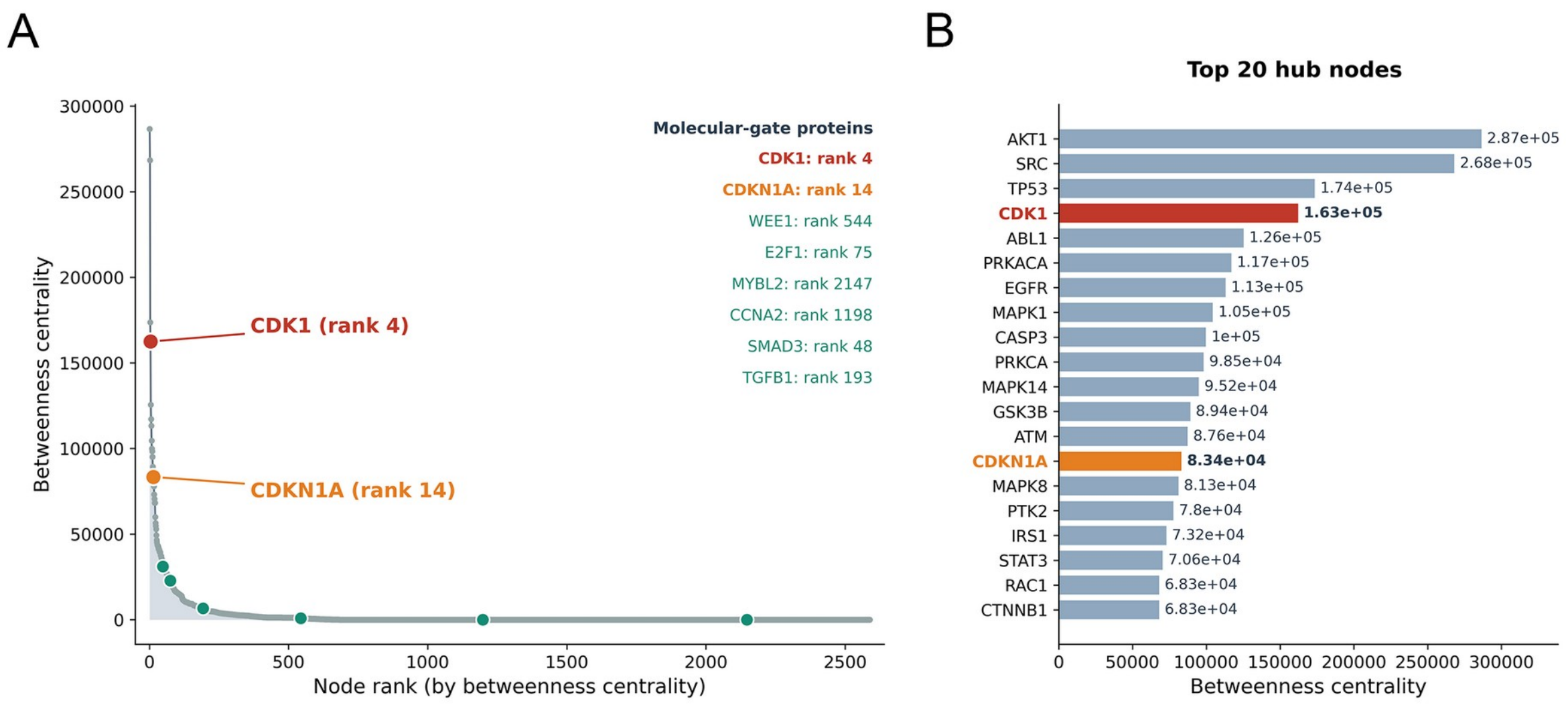

Supplementary Figure 9. Betweenness centrality across the dynamic regulatory network identifies CDK1 as a central bottleneck.

Betweenness centrality was computed for all nodes of the reconstructed regulatory network at a representative pseudotime bin of Trajectory 3 (GEM + TGF-β1), the trajectory associated with chemoresistance. (A) Rank-ordered betweenness across all 2,588 network nodes. The distribution is strongly heavy-tailed: a small number of hub nodes carry disproportionately high betweenness, while the majority of nodes contribute little to global information flow. The eight molecular-gate proteins of the CDK1–CDKN1A–WEE1 bottleneck (Supplementary Figure 10) are highlighted (green), with CDK1 (rank 4) and its opposing inhibitor CDKN1A (rank 14) marked as outliers at the high-betweenness end of the distribution. (B) The twenty highest-ranked hub nodes by betweenness centrality. Among the gate proteins, only CDK1 (red) and CDKN1A (orange) fall within the top twenty; CDK1 ranks among the top hubs of the entire network, placing the CDK1–CDKN1A checkpoint at a topologically privileged control point through which convergent S-phase and TGF-β1 signals must pass.

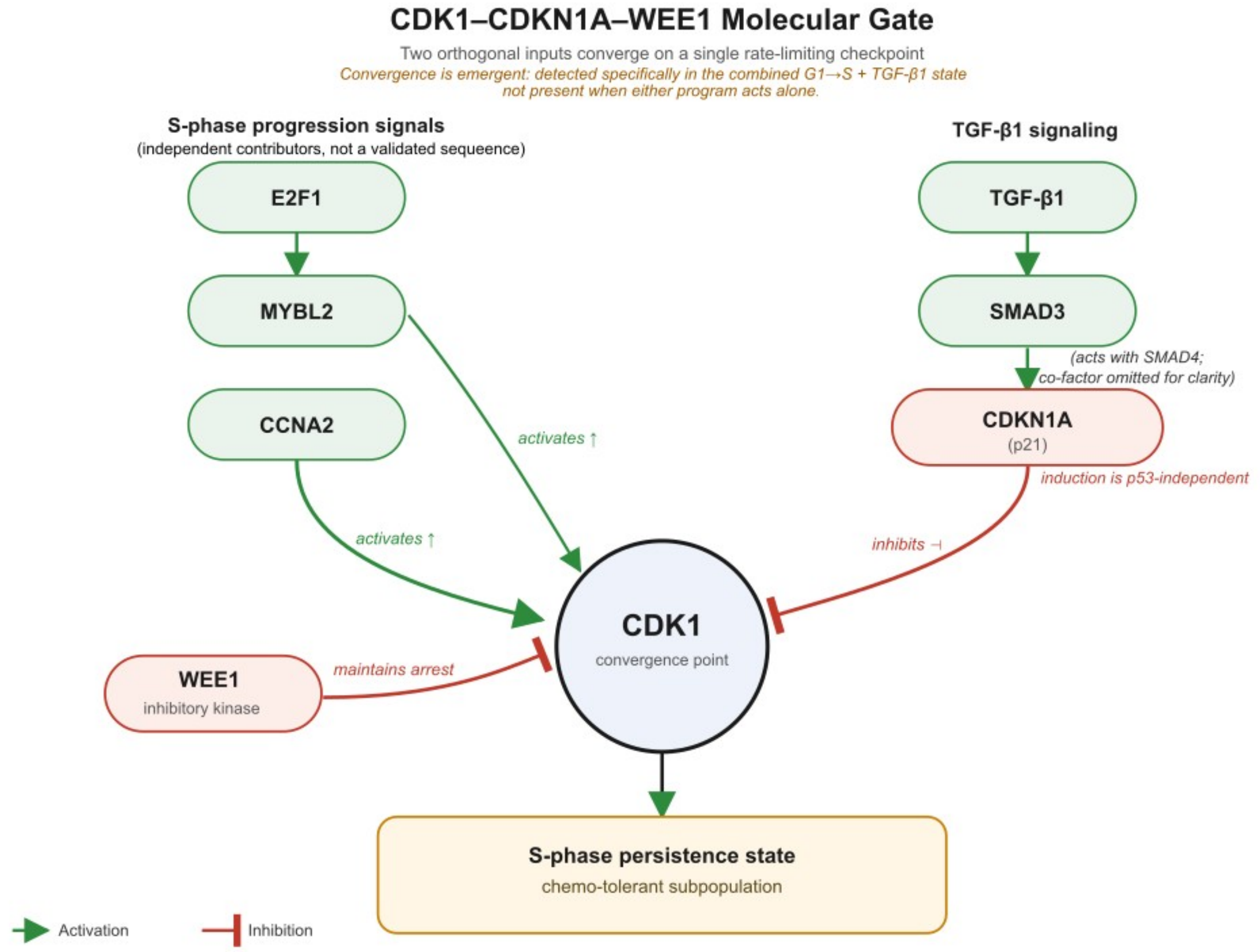

Supplementary Figure 10. Molecular gate schematic of the CDK1–CDKN1A–WEE1 bottleneck.

Two orthogonal inputs converge on CDK1, the rate-limiting checkpoint of the S-phase persistence state. S-phase progression signals (E2F1, MYBL2 and CCNA2) activate CDK1, whereas TGF-β1 signalling through SMAD3 induces the cyclin-dependent kinase inhibitor CDKN1A (p21) independently of p53. CDKN1A and the inhibitory kinase WEE1 both repress CDK1, maintaining S-phase arrest. This convergence is emergent, arising only in the combined G1→S + TGF-β1 state (Fig. 4), and defines an S-phase-persistent, chemotolerant subpopulation underlying gemcitabine resistance. Green arrows denote activation; red bars denote inhibition.

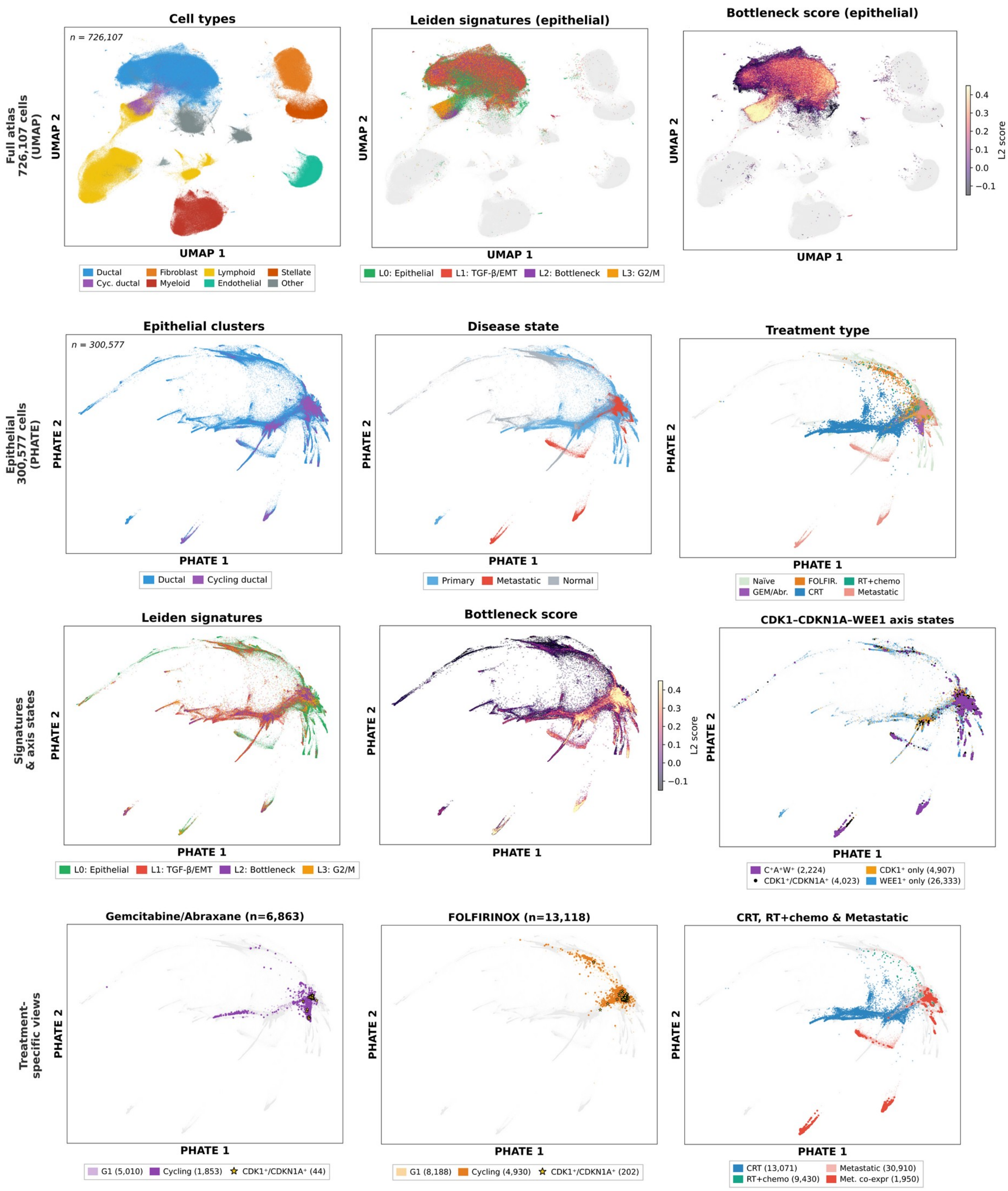

Supplementary Figure 11. Multi-level mapping of CDK1–CDKN1A–WEE1 bottleneck signatures in the PDAC single-cell atlas. (A–C) Full atlas view (726,107 cells, 231 patients, 12 studies). (A) UMAP embedding colored by cell-type annotation, showing the major compartments: ductal (blue), cycling ductal (purple), fibroblast (orange), myeloid (red), lymphoid (yellow), endothelial (teal), stellate/pericyte (dark orange), and acinar/endocrine (grey). (B) Leiden signature assignment projected onto the full atlas (epithelial cells only; non-epithelial in light grey). Cells are colored by the dominant signature score among L0 (epithelial baseline, green), L1 (TGF-β/EMT, red), L2 (S-phase + EMT bottleneck, purple), and L3 (G2/M proliferative, orange). (C) Continuous Leiden 2 (bottleneck) score overlaid on epithelial cells (magma colormap). (D–F) Epithelial subset (300,577 cells) visualized by PHATE embedding, which preserves continuous trajectory structure. (D) Cluster annotation distinguishing ductal and cycling ductal populations. (E) Disease-state mapping: primary tumors (blue), metastatic lesions (red), and normal/donor tissue (grey). (F) Treatment-type overlay showing the distribution of chemotherapy survivors:

gemcitabine/abraxane (purple), FOLFIRINOX (orange), chemoradiation/CRT (blue), RT + chemotherapy (teal), combined FOLFIRINOX + gemcitabine/abraxane (dark yellow, where present), and metastatic (pink). Naïve cells are shown in pale green as background. (G–I) Bottleneck signature and axis-state mapping on epithelial PHATE. (G) Dominant Leiden signature assignment. (H) Continuous Leiden 2 (bottleneck) score. (I) CDK1–CDKN1A–WEE1 combinatorial axis states: triple-positive cells (C+A+W+, n = 2,224; purple), CDK1+/CDKN1A+ co-expressing cells (black), CDK1+-only cells (orange), and WEE1+-only cells (blue). (J–L) Treatment-specific views. (J) Gemcitabine/abraxane survivors (n = 6,863) with G1-arrested cells in light purple and cycling cells in dark purple; CDK1+/CDKN1A+ co-expressing cells are highlighted as yellow stars. (K) FOLFIRINOX survivors (n = 13,118), same color scheme in orange tones. (L) Remaining treatment groups and metastatic cells: CRT (blue), RT + chemotherapy (teal), and metastatic cells (pink) with CDK1+/CDKN1A+ co-expressing metastatic cells in dark red.

Supplementary Note 1. Evaluation of systemic single-cell analysis approaches

Single-cell transcriptomics provides a powerful window into the vast state space of a cellular population. As an addition to relying on robust bulk RNA-seq analysis[1], which captures an averaged expression profile, single-cell data can resolve myriad intermediate states—some closer, others farther from a given attractor—and the continuous transitions between these states. In our 3D tissue model, such granularity is especially beneficial for dissecting the dynamic behavior of cells under different treatments, as it enables detailed observation of stochastic variations and transitional subpopulations that bulk methods would obscure.

By applying methods such as Leiden clustering, we visualize the heterogeneity of conditions across ~10,000 individual cells, identifying major attractors (e.g., G1→→S1, G2–M clusters, G1+TGF-β1, S+TGF-β1) and "bridge" cells that lie between them. Trajectory analyses then enable us to reconstruct the principal paths by which cells transition from one state to another. This approach also uncovers parallel transitions—such as those induced by TGFβ-1 from distinct "root" states—highlighting how multiple, seemingly separate lineages can converge under a shared state change "pressure." Importantly, we can track the earliest pseudotime phases of these trajectories to identify potential early markers of aggressiveness or therapy resistance, which may hold clinical relevance.

In addition to clustering cells, we can cluster genes by their co-expression patterns along these trajectories, revealing regulatory cascades. For instance, TGFβ-driven EMT involves a stepwise activation of downstream genes (e.g., TGFBI, SPOCK1, PMEPA1), each turning on at slightly different times, thus grasping the cascading effect in causalities that are separated in time. Likewise, pathway or gene set enrichment analyses (GSEA) use prior knowledge to capture the cascading effect. Classic static methods—such as differentially expressed genes (DEGs) under specific conditions or principal component analysis (PCA)—lack temporal resolution. For example, PFA highlights statistically informative genes, such as SPOCK1 and TGF-B1 (EMT

pathway), suggesting their statistical independence. This would only be true in a static world, but it would overlook the temporal dependencies inherent in biologically orchestrated pathways. SPOCK1 and TGF-β1 are clearly statistically connected, as one expression will ultimately cause the expression of the other (even if hours separate the correlation). This is a very old and common problem in analyzing gene co-expression networks[2], and a reason why they are no longer used as a metric for statistical dependence or independence. Using the NES (Normalized Enrichment Score from GSEA) here as a metric is a more reliable measure of statistical independence.

In summary, the combination of single-cell clustering (Leiden), pseudotime inference, trajectory reconstruction, gene set enrichment, and network analysis provides a complementary toolkit that captures both static 'snapshots' and dynamic transitions, enabling the construction of a systems-level decision-point map. Crucially, this approach also accounts for network dynamics: as gene expression continuously changes, cells update their regulatory logic, resulting in signaling behaviors that differ markedly across time points or cell states. By integrating expression trajectories with dynamic network changes, we can uncover the root causes of cascading gene-expression effects, even when these occur in separate time or space. This multi-angle framework is essential for a systems-level understanding—encompassing cascading effects, logically connected events over time, and branching lineage outcomes. For immediate clinical applications, such as rapid biomarker discovery, simpler static methods like DEGs or PCA may suffice due to their accessibility and interpretability. However, for fundamental research aimed at building mechanistic models or pinpointing early drivers of state transitions, deeper single-cell approaches are indispensable. Our study demonstrates how integrating these methods yields a comprehensive understanding of system behavior, providing critical insights into the biology of drug resistance and tumor progression.

Supplementary Note 2. Tissue surface and core

Determining whether the gene-expression patterns we observe correspond to cells located at the tumor surface or core will ultimately be answered in future after we can obtain spatial transcriptomics data or analyse dissections e.g. by LASER catapulting experiments. However, currently there is scarcity of spatial transcriptomics datasets[3].

Nonetheless, certain clues suggest a possible connection between cell-cycle activity and localization. For instance, MKI67, enriched in Leiden clusters 2 and 3, is not only a robust proliferation marker but has also been associated with leading-edge cells[4].

Recent spatial mapping of pancreatic tumors[3] found metastatic niches exhibit enrichment in E2F targets, mTOR activation, and glycolysis, along with reduced G2–M activity. These patterns parallel our findings (e.g., reduced AURKA activity in cells undergoing EMT, as indicated in Supplementary Figure 4); both systems highlight the interplay between cell-cycle regulation and tumor microenvironmental cues.

Future work, leveraging integrative spatial omics approaches or direct in situ gene tagging will even better illuminate how localized niches within the tumor contribute to differential chemoresistance, thereby guiding future therapeutic strategies.

Supplementary References

1. Caliskan, A. et al. Mathematical strategies for predicting resistant subpopulations from scRNAseq data of a PANC-1 3D tissue model: Insight into gemcitabine resistance and TGFB1-induced invasion and EMT. Comput. Struct. Biotechnol. J. 27, 4476–4495 (2025).
2. Serin, E. A. R., Nijveen, H., Hilhorst, H. W. M. & Ligterink, W. Learning from co-expression networks: possibilities and challenges. Front. Plant Sci. **7**, 444 (2016).
3. Khaliq, A. M. et al. Spatial transcriptomic analysis of primary and metastatic pancreatic cancers highlights tumor microenvironmental heterogeneity. Nat. Genet. **56**, 2455–2465 (2024).
4. Uxa, S. et al. Ki-67 gene expression. Cell Death Differ. 28, 3357–3370 (2021).
